# Bio-Based Coatings: Progress, Challenges and Future Perspectives

**DOI:** 10.3390/polym17243266

**Published:** 2025-12-09

**Authors:** Lijian Xia, Taijiang Gui, Junjun Wang, Haoyuan Tian, Yue Wang, Liang Ning, Lianfeng Wu

**Affiliations:** State Key Laboratory of Coatings for Advanced Equipment, Marine Chemical Research Institute Co., Ltd., Qingdao 266072, China; xialijian@sinochem.com (L.X.); guitaijiang@sinochem.com (T.G.); wangjunjun01@sinochem.com (J.W.); tianhy0531@163.com (H.T.); wangyuemoo@163.com (Y.W.); ningliang@sinochem.com (L.N.)

**Keywords:** bio-based coatings, sustainable polymers, biomass conversion, circular economy

## Abstract

In response to environmental concerns and the depletion of fossil resources, transitioning coatings toward sustainability is imperative. Bio-based coatings, derived from renewable biomass, represent a highly promising development pathway. This review comprehensively summarizes recent advances, prevailing challenges, and future prospects of bio-based coatings, with a focus on bio-based polymer resins—serving as the primary film-forming materials—and key auxiliary components such as pigments and fillers, additives, and solvents. This review systematically elaborates on the definition of bio-based coatings, their raw material sources, and international standards for bio-based carbon content determination. The core strategies for converting biomass into coating components are critically analyzed, namely direct utilization, physical blending, chemical modification, and biosynthesis. Furthermore, the synthesis, properties, and applications of key bio-based polymer systems—including epoxy, polyurethane, alkyd, and acrylic resins—are critically discussed, with particular emphasis on how molecular engineering enhances their performance and functionality. Despite significant progress, bio-based coatings still face several challenges, such as balancing performance and cost, ensuring the stability of raw material supply chains, and establishing globally unified standards. This review concludes that the integration of chemical modification and biosynthesis technologies, coupled with the establishment of a unified bio-based content standard system, constitutes two core drivers for advancing bio-based coatings from “green alternatives” toward “high-performance dominance” in the future.

## 1. Introduction

Against the escalating threat of global warming, governments and international bodies worldwide have proactively enacted policies to curb carbon dioxide emissions. This impetus drives green technology innovation and enhances the global competitiveness of industries and economies, firmly establishing green sustainability as the cornerstone of future development [1].

In the chemical industry, sustainability manifests across several pivotal dimensions, including minimizing volatile organic compound (VOC) emissions, optimizing operational efficiency, reducing toxicity, decreasing energy consumption, minimizing waste, enhancing recyclability, extending product durability, and incorporating renewable feedstocks [2]. These advancements are propelled by significant technological breakthroughs, enabling the development and seamless integration of innovative solutions. Prominent examples of environmentally friendly coatings embodying these principles are water-borne, radiation-curable, solvent-free high-solid-content, and powder coatings [3].

Since the mid-20th century, Europe and North America have made substantial strides in the research and development of eco-friendly coating technologies. Their endeavors have predominantly centered on advancing the production and commercialization of sustainable coating variants, with the overarching objective of mitigating environmental impact while simultaneously maintaining or enhancing product performance. For instance, solvent-free polyurethane coatings, valued for their zero VOC emissions and chemical resistance, are widely used in the automotive industry. Similarly, high-solid-content coatings, which reduce solvent use via lower molecular weight resins, have become a mainstay in industrial painting. China began introducing and localizing these technologies in the 1990s, later than its developed counterparts.

With increasingly stringent environmental policies and evolving market demands, products like waterborne and UV-curable coatings experienced rapid growth after 2000. However, a significant limitation remains: most of these coatings still rely heavily on petroleum-derived chemicals, which face challenges of reserve depletion and adverse environmental impacts from their extraction and use [3,4,5]. This lingering dependency on fossil resources has catalyzed the emergence of bio-based coatings, derived from renewable resources like plant oils, fatty acids, cellulose, cardanol, lignin, and other agricultural by-products. Regarded as a viable and sustainable alternative to petroleum-based chemicals, bio-based coatings are garnering significant attention in both academic and industrial circles [3,6]. The market for these coatings is forecasted to grow substantially, projected to reach USD 29.4 billion by 2032, driven by product sustainability and alignment with green consumerism trends [7]. Bio-based coatings are already being applied in diverse fields such as construction [8], furniture [9], automobiles [10], textiles [11], and packaging [12], demonstrating their broad application potential. A landmark development was recently announced by Chugoku Marine Paints and Mitsui Chemicals: a bio-based epoxy resin coating, CMP NOVA 2000 (Bio), has been selected for a liquefied ammonia tanker scheduled for delivery in 2026. This coating uses ISCC PLUS-certified bio-based epoxy resin and reduces carbon dioxide emissions via the mass balance approach. While commercial bio-based coating applications in shipbuilding were previously limited to the “experimental stage” due to cost and technical maturity challenges, this project represents the world’s first use of a bio-based epoxy coating on ships [13,14], signaling immense market potential for bio-based coatings in the shipbuilding industry and beyond.

Preliminary research efforts have identified key innovative technologies driving the development of bio-based coatings. These span the synthesis of bio-based resins, the development of bio-based pigments, fillers, additives, and solvents, as well as the optimization of coating formulations. Collectively, these core technologies lay the groundwork for advanced bio-based coating types tailored to diverse specifications. Given the growing global demand for sustainable solutions, this review provides an in-depth summary of recent research, focusing on enhancing performance and broadening the application spectrum of bio-based coatings across various industrial sectors.

## 2. Definition, Classification, and Global Context of Bio-Based Coatings

### 2.1. Definition and Evaluation Criteria of Bio-Based Coatings

As the name suggests, bio-based coatings refer to coatings synthesized using biomass feedstocks. Broadly defined, biomass [15] includes organic materials produced through photosynthesis, encompassing all living organisms (e.g., animals, plants, microorganisms) and their derivatives—these materials are specifically used as renewable raw materials for formulating coatings. Among relevant standards, the precise definition and scope of biomass are clearly specified in China’s national standard GB/T 39514 [16]; while ASTM D6866-24a [17] does not define the definition or scope of biomass, it plays a pivotal role in determining bio-based carbon content. This method quantifies bio-based carbon content by measuring the radiocarbon isotope (^14^C), with test results expressed as “% bio-based carbon”. A 100% result indicates that all carbon originates from contemporary biological sources (e.g., plants, animals), while 0% signifies that all carbon is derived from fossil sources (e.g., petroleum, coal); values between 0% and 100% represent a mixture of both [18]. This method has become a globally recognized benchmark for bio-based content evaluation, laying the foundation for the development of regional standards.

In the United States, the U.S. Department of Agriculture (USDA) “Bio-Preferred Program” exerts significant influence on bio-based carbon content standards for coatings. To obtain USDA certification, bio-based coatings must meet a minimum bio-based carbon content of 25%. For bio-based products in Europe, relevant regulatory requirements are supplemented by the EN 16640 standard [19]. Specifically, bio-based coatings for the construction sector are required to be tested in accordance with EN 16640 [18], which sets a minimum bio-based carbon content of 30%.

Although China has not yet issued a nationally unified standard for bio-based carbon content in coatings, the domestic coating industry has not fallen into a passive waiting mode; instead, it is proactively advancing the development of the bio-based coating industry through strategic initiatives. To fill this standard gap, the China National Coatings Industry Association (CNCIA) has taken the lead in collaborating with relevant enterprises to pioneeringly develop industrial association standards that align with international norms. The core objective of these standards is to improve the technical system for bio-based coatings and provide support for the standardized development of the industry. For instance, the CNCIA has released T/CNCIA 01032-2024 [20]. This key association standard specifies the testing methods and minimum thresholds for the bio-based content of alkyd resin coatings.

### 2.2. Classification of Bio-Based Coatings by Raw Materials Source

Generally speaking, coatings are mainly composed of components such as film-forming resins, pigments and fillers, additives, and solvents, and bio-based coatings are no exception. Therefore, from the perspective of raw material sources, it is not only feasible to carry out exploration and research and development work on each component of bio-based coatings but also the results are remarkable, which can support a huge industrial system.

Bio-based coating materials, derived from renewable biomass via biological, chemical, or physical valorization, are inherently environmentally friendly, biodegradable, and sourced from sustainable feedstocks [21]. Therefore, they can be classified into plant sources (plant/vegetable oils [22], plant phenols [23], polysaccharides and derivatives [24,25,26], rosin [27,28], biological acids [29,30,31,32,33], bio-non-isocyanate [34,35,36]), animal sources (chitin [37,38], chitosan [37,38,39,40], collagen [41]), and microbial sources [42,43,44,45]. Table 1 presents a comparison of the characteristics of bio-based materials with different raw material sources.

### 2.3. Global Research Status and Data on Bio-Based Coatings

#### 2.3.1. Publication Trend

According to Web of Science data, the number of annual publications on bio-based coatings has increased from 52 (2010) to 876 (2024), with a compound annual growth rate (CAGR) of 18.2%. China, the United States, and Germany are the top three contributing countries.

#### 2.3.2. Industrial Market Data

Market size: the global bio-based coatings market was valued at USD 15.2 billion in 2024 and is projected to reach USD 29.4 billion by 2032 (CAGR 9.75%) [7].

Regional distribution: Europe dominates the market due to strict environmental policies, followed by Asia-Pacific with rapid growth driven by China’s “dual-carbon” strategy; key application sectors: construction, packaging, and automotive are the top three application fields.

## 3. Bio-Based Materials Conversion Strategies

### 3.1. Utilize Directly

A range of biomass materials (e.g., natural resins, oils, and adhesives) can be directly utilized for coating preparation through straightforward physical processing methods—including dissolution, heating, grinding, and mixing—thus avoiding the requirement for intricate chemical modifications (e.g., esterification, etherification, polymerization, etc.).

**Figure 1:**
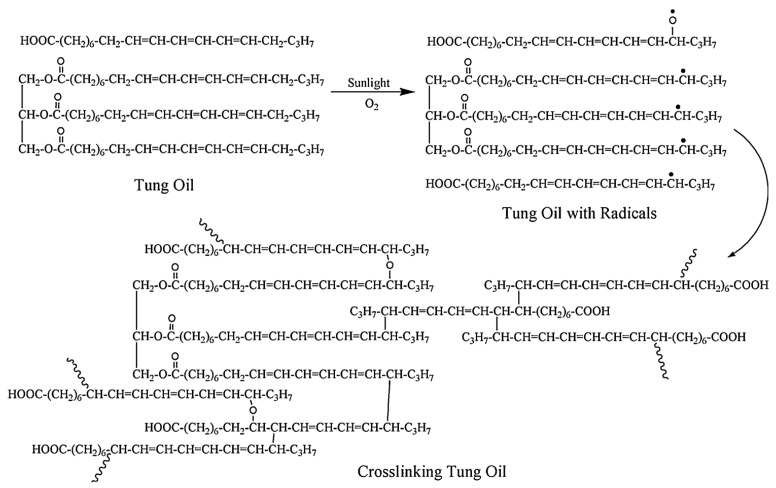
The drying mechanism of tung oil [47]. Copyright 2019, Elsevier.

Tung oil, extracted from the nuts of the tung tree (*Vernicia fordii*), primarily comprises fatty acids dominated by conjugated trienes. These conjugated triene structures enable significantly faster polymerization compared to non-conjugated double bond systems—such as those found in linseed oil [46]. As a classic drying oil, tung oil cures upon exposure to air, forming a transparent, continuous film; the mechanism of film formation through curing is depicted in Figure 1. Notably, it exhibits excellent water-repellent properties, which remain effective even after prolonged severe aging and weathering. Currently, tung oil is utilized to enhance wood quality [47].

**Figure 2:**
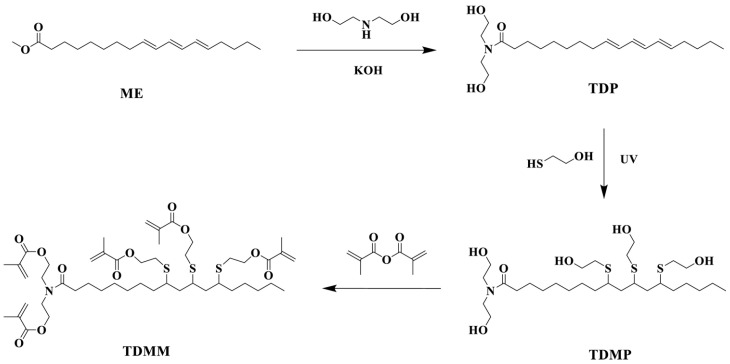
Synthesis route of tung–oil-based acrylate (TDMM) [52]. Copyright 2021, Elsevier.

For instance, Humar and Lesar [48] emphasized its dual functionality: it provides reliable protection against both brown-rot and white-rot fungi while also preventing wood from absorbing water over short-, medium-, and long-term periods. It should be noted that a key limitation of pure tung oil is its relatively long drying time (typically exceeding five days), which restricts its broader application in industrial contexts. Based on the aforementioned challenges, the development of tung oil in recent years has focused on two main approaches: either the independent synthesis of tung oil-based derivatives or their co-synthesis with other monomers (Figure 2). These strategies aim to produce bio-based monomers compatible with photopolymerization, which are then used to fabricate bio-based photopolymer coatings. This advancement has significantly enhanced both the curing efficiency and overall performance of tung oil-derived materials [49,50,51,52].

Beyond the materials mentioned previously, other biomass materials such as beeswax [53], shellac [54], and starch [55] are also viable options for directly forming coating films. For example, beeswax can be used to create protective coatings, which are beneficial for fruit and vegetable preservation by reducing moisture loss and inhibiting microbial growth.

### 3.2. Physical Blending

Despite their considerable potential benefits, bio-based materials still face several key obstacles—most notably poor miscibility, processability challenges, and significant disparities in physical properties—when compared to their conventional counterparts [56]. Meanwhile, some functional additives such as compatibilizers and plasticizers, as well as some biogenic functional fillers, can all be mixed together to enhance the performance of the polymer [57]. Polymer blending is a widely used strategy that enables extensive adjustment of material properties. Bio-based polymeric blends, which incorporate two or more polymers derived from biological sources, offer a cost-effective alternative to the development of entirely new monomers or polymers, thereby reducing research and development expenses. Such blends can be rapidly adapted to meet evolving technological requirements and provide combinations of properties that are often difficult to achieve with novel polymer structures.

**Figure 3:**
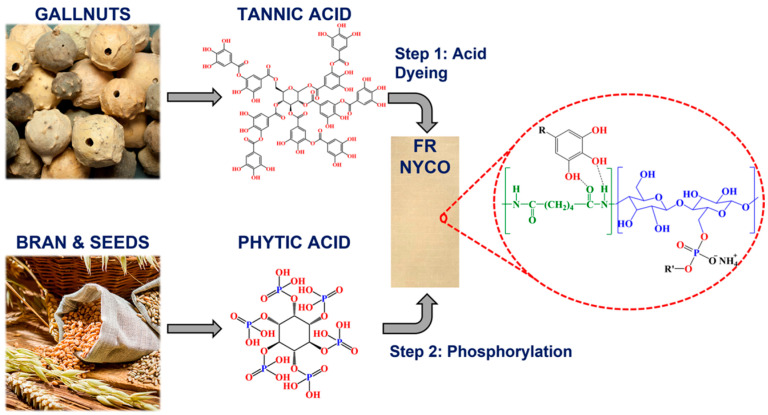
Schematic showing the source of TA and PA along with their chemical structures and the notional bonding hypothesis [60]. Copyright 2021, American Chemical Society.

Biomass polymers are mixed together to enhance the performance of coatings, and are widely used in fields such as anticorrosion [58], heat insulation [59], flame retardancy [60], and wear resistance [61]. Lv and colleagues [58] synthesized bio-based materials derived from cardanol/cardol, subsequently blended with a commercial bisphenol A diglycidyl ether epoxy resin (DGEBA) for use in anticorrosion coatings. As shown in Figure 3, Kulkarni and co-workers [60] employed a synergistic bio-based system comprising tannic acid (TA) and phytic acid (PA) to impart flame-retardant properties to nyco fabric. TA and PA were sequentially deposited onto the nylon and cotton fibers via hydrogen bonding and phosphorylation, respectively. The results demonstrated that the flame retardancy is achieved primarily through enhanced char formation facilitated by the combined action of TA and PA. This TA–PA system exhibits significant potential as an effective and sustainable bio-based flame-retardant treatment for textile applications.

To develop materials with potential packaging applications, bio-based poly(lactic acid)/poly(3-hydroxybutyrate) (PLA/PHB) blends were melt-blended with a natural hydrophobic plasticizer, glyceryl tributyrate (TB). The influence of TB on the material properties was systematically investigated. Mechanical testing revealed a progressive increase in elongation at break with higher plasticizer content. Among the formulations evaluated, the blend containing 15 wt% TB exhibited the most suitable combination of properties for film manufacturing, including enhanced toughness and ductility, good water barrier performance, and transparency with a slight amber tint [62].

As the second most essential component in coating formulations after the resinous film-forming material, functional fillers impart special properties to the coating film. In the field of fireproof coatings, for instance, intumescent coatings offer an optimal balance of safety, aesthetics, cost-effectiveness, and ease of application, establishing them as the dominant technology in modern building fire protection. These coatings incorporate varying proportions of industrial fillers—such as titanium dioxide (TiO_2_) and aluminum hydroxide (Al(OH)_3_)—and/or bio-based fillers, such as eggshell and rice husk ash. Test results demonstrate that coatings containing bio-based fillers exhibit significantly enhanced fire resistance. Owing to the highly efficient flame-retardant performance of coatings with mixed industrial and bio-based fillers, their use represents a practical and promising approach to meet fire protection requirements, control fire development, and inhibit fire spread [63,64].

### 3.3. Chemical Modification

Chemical modification is the most widely applied and studied core approach, thanks to its unique advantages in precise molecular structure regulation and performance boundary expansion. Its essence lies in the “tailoring and reconstruction” of bio-based molecular structures via chemical reactions. This process involves introducing new functional groups or altering the linkage patterns of molecular chains, ultimately endowing materials with a range of properties they inherently lack—including processing properties (such as melt fluidity), mechanical properties (such as strength and elasticity), and functional characteristics (such as water resistance, biodegradability, and thermal stability).

**Figure 4:**
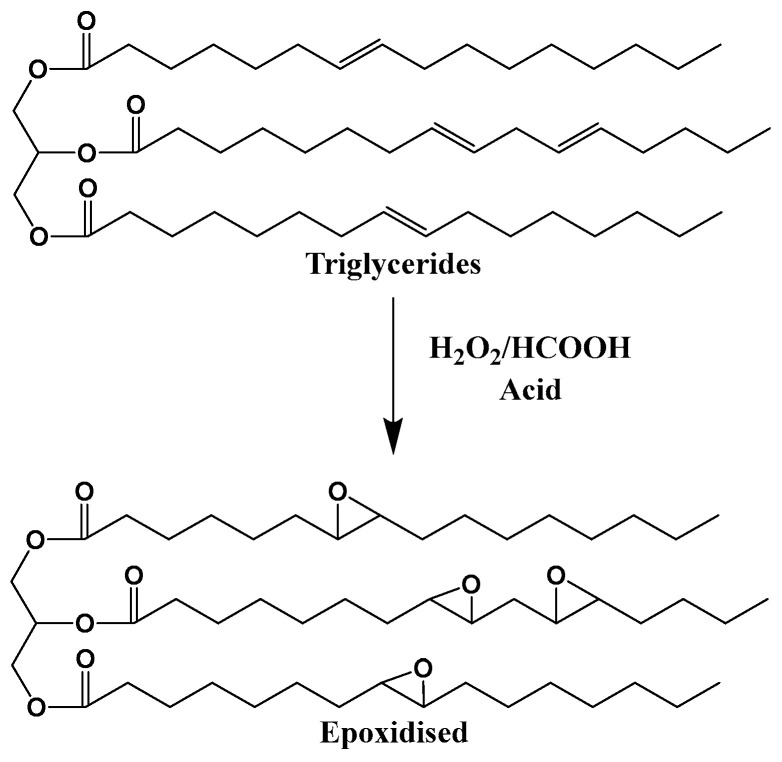
Epoxidation of triglycerides [65]. Copyright 2024, Wiley.

**Figure 5:**
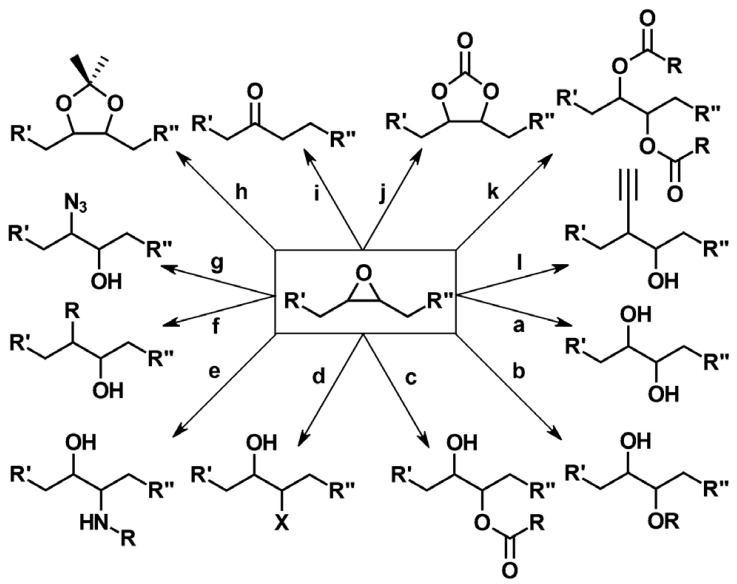
Selected ring opening reactions of fatty epoxides to give: (**a**) vicinal diol, (**b**) alkoxy-hydroxy, (**c**) acyloxy-hydroxy, (**d**) halo-hydroxy (X = Cl, Br, I, or F), (**e**) alkylamino-hydroxy, (**f**) alkyl-hydroxy or aryl-hydroxy, (**g**) hydroxy-azide, (**h**) acetonide (ketal), (**i**) ketone, (**j**) carbonate, (**k**) diacyloxy, and (**l**) alkynyl-hydroxy products [67]. Copyright 2022, Wiley.

Plant oil is one of the renewable resources for preparing bio-epoxy resins, and its main component is various triglycerides. As shown in Figure 4, the carbon–carbon double bonds contained in triglyceride molecules are usually the structural basis for various chemical modifications of vegetable oils. However, these carbon–carbon double bonds have relatively low reactivity, requiring chemical modification to enhance the overall reactivity of triglycerides. Epoxidation is a chemical process that is easy to operate and has mature research, which can effectively solve this problem: it can convert the carbon–carbon double bonds in triglycerides into ethylene oxide rings (oxirane rings), thereby endowing triglycerides with epoxy functional groups and ultimately significantly improving their reactivity [65,66]. Oxiranes (epoxy groups) are highly susceptible to ring-opening, particularly in acidic environments. Their inherent reactivity gives rise to a rich diversity of reactions, as illustrated in Figure 5 [67].

**Figure 6:**
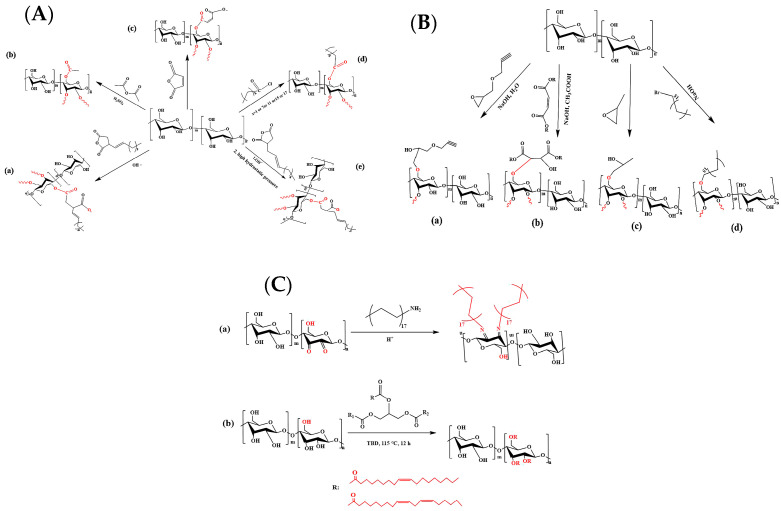
(**A**) Schematic diagram of esterification reaction. (**B**) Schematic summary of etherification. (**C**) Schematic summary of nucleophilic reaction (**a**) and transesterification (**b**) [72]. Copyright 2020, Elsevier.

Starch, a renewable polysaccharide, has garnered considerable research attention due to its low cost, excellent film-forming ability, biocompatibility, abundance, renewability, and biodegradability. Nevertheless, the inherent hydrophilicity, brittleness, sensitivity to enzymes and acids, insolubility in organic solvents, etc., of native starch significantly restrict its industrial applicability [68]. To address this limitation, as shown in Figure 6, recent advances in chemical modification strategies including esterification [68], etherification [69], crosslinking [70], grafting [69], and condensation reactions [71], among others, are designed to improve the thermal stability and hydrophobicity of starch [72].

After chemical modification, the overall properties of starch—including its thermal stability, hydrophobicity, and antibacterial performance—are markedly enhanced. As a result, chemically modified starch exhibits great potential for application in fruit and vegetable preservation coatings [73], edible films [74], and packaging coatings [75].

**Figure 7:**
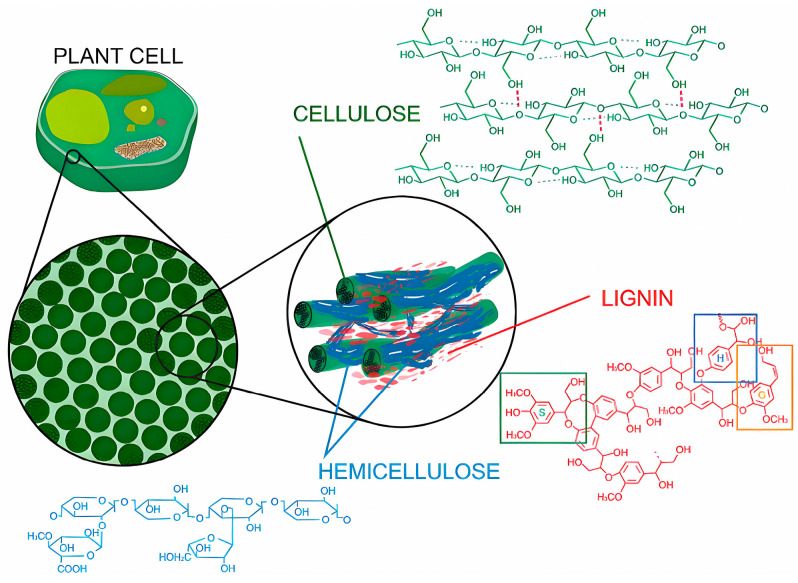
Composition of lignocellulosic biomass and the structural roles of cellulose, hemicellulose, and lignin. Lignin is mainly composed of three structural units: syringyl (S, highlighted in the green box), guaiacyl (G, highlighted in the red box), and p-hydroxyphenyl (H, highlighted in the blue box). [76] Copyright 2023, Royal Society of Chemistry.

Lignin, a polyphenol-rich natural polymer obtained as a by-product of the wood-pulping process, is a major constituent of the lignocellulosic matrix in which it is intimately associated with cellulose and hemicellulose (Figure 7). It is one of the most abundant polyphenolic compounds and the second largest renewable biomass resource after cellulose.

On one hand, lignin and its derivatives exhibit prominent hydrophobicity and barrier properties, making them suitable as raw materials for coatings [77]. On the other hand, lignin contains chromophoric functional groups that enable it to absorb broadly across the UV spectrum (250–400 nm). Consequently, its incorporation as a natural ingredient in sunscreen creams, transparent films, paints, varnishes, and antimicrobial coatings has been extensively studied. Both native and modified lignin enhance UV protection when blended with other materials [78,79,80].

**Figure 8:**
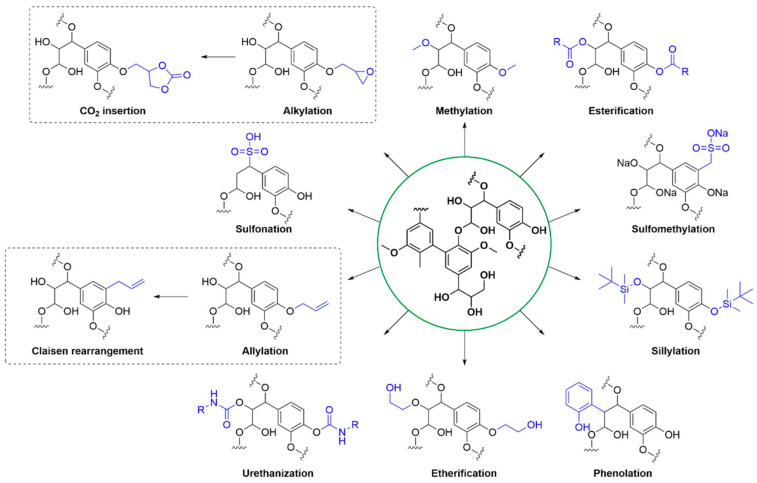
Examples of chemical modifications of technical lignin [76]. Copyright 2023, Royal Society of Chemistry.

Furthermore, studies have reported that the final lignin structure contains various functional sites, most notably phenolic and aliphatic hydroxyl groups, carboxylic acids, and methoxy groups [81]. The abundance of reactive surface hydroxyl and carboxyl groups in lignin endows it with significant potential for tailored chemical modification—by introducing various functional groups—to suit a wide range of applications [82], which facilitates the value-added utilization of lignin. As shown in Figure 8, lignin has been modified using numerous techniques, including esterification, phenolation, and etherification [76].

**Figure 9:**
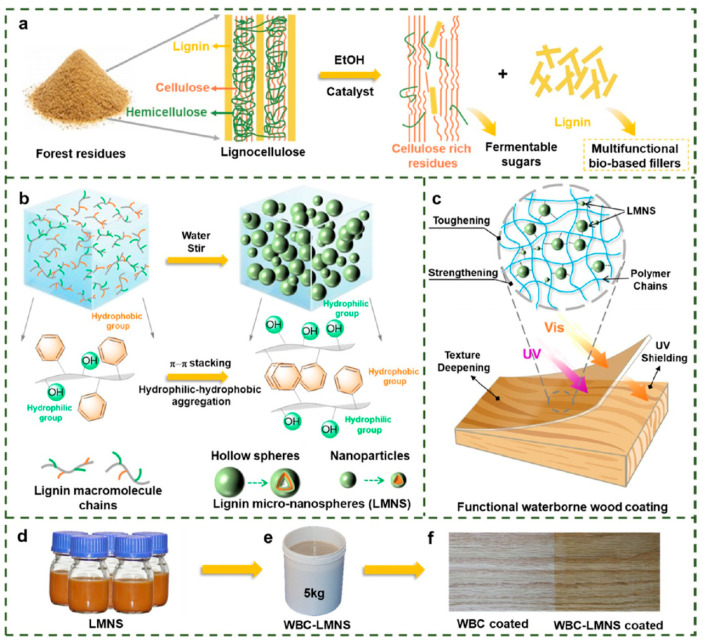
Schematic diagram of lignin valorization to colloidal LMNS used as multifunctional bio-based fillers for WBC enhancement. (**a**) Ethanosolv lignocellulose fractionation to CRR and lignin occurs in one pot, and the lignin is valorized to multifunctional bio-based fillers for WBC. (**b**) Schematic showing LMNS preparation via self-assembly. The hollow lignin microspheres and nanospheres are obtained simultaneously via π–π stacking and hydrophilic–hydrophobic aggregation of lignin macromolecules. (**c**) Schematic showing the LMNS-reinforced WBC on a wood substrate. The LMNS toughens and strengthens the polymer matrix and endows the WBC with UV-shielding and texture-deepening performance. Photographs of (**d**) stable LMNS suspensions, (**e**) LMNS-reinforced WBC (5 kg), and (**f**) finishing a wood substrate with neat WBC and LMNS-reinforced WBC, respectively [85]. Copyright 2022, American Chemical Society.

SAL additives were synthesized via an esterification reaction between the hydroxyl groups on the lignin surface and succinic anhydride (SAN). The incorporation of SAN imparted a plasticizing effect to lignin, leading to a gradual decrease in the glass transition temperature (Tg). Compared to the crosslinked coating derived from pure lignin, the SAL-based polyethylene (PE) coating after crosslinking exhibited enhanced thermal stability, film-forming capability, solvent resistance, dynamic surface hardness, and hydrophobicity. At the same time, it maintained high adhesion strength on various substrates [83]. Lignin was esterified with tall oil fatty acids and applied as a coating onto fiber-based packaging materials. This treatment led to a significant reduction in both water vapor and oxygen permeability. The resulting lignin–fatty acid ester coating shows great potential as a sustainable alternative to conventional petroleum-based barrier materials [84]. As shown in Figure 9, Song and their teammates [85] reported that organosolv lignin generated from biorefineries was valorized to colloidal lignin micro-nanospheres (LMNSs) prepared by a facile self-assembly process and applied as a multifunctional bio-based filler for waterborne wood coating (WBC). The incorporation of an LMNS in WBC strengthened and toughened the polymer matrix without impairing its transmittance. In addition, adding the LMNS highlighted the color and texture of wood and improved the discoloration resistance of the WBC-coated wood based on blocking of UV rays. The present study provided a new strategy for lignin valorization that could allow the sustainable production and utilization of lignin and advances the development of multifunctional WBC.

Cellulose, a pivotal linear polysaccharide composed of β-D-glucose units linked by (1-4)-glycosidic bonds, serves as a fundamental structural component in the primary cell walls of green plants. It coexists with other organic constituents—such as lignin, hemicellulose, pectin, and proteins—and stands as the most abundant natural polymer materials in nature. The polymer chain of cellulose comprises numerous hydroxyl (-OH) groups, which enable the formation of extensive intramolecular and intermolecular hydrogen bonds. These robust hydrogen bonds endow cellulose with high crystallinity and rigidity. By contrast, the amorphous regions, characterized by weaker hydrogen bonding, contribute to its hydrophilicity, flexibility, and structural accessibility. Collectively, the high density of hydroxyl groups and the resultant hydrogen-bonding network dictate the essential properties of cellulose, including its hydrophilicity, bio-degradability, chirality, and chemical reactivity [86].

It is commonly understood that cellulose is primarily extracted from higher plants such as cotton, wood, straw, flax, and agricultural waste [87]. However, cellulose can also be biosynthesized by other organisms. For instance, tunicates represent a specific group of animals capable of synthesizing cellulose endogenously, a substance often referred to as tunicin [87]. Similarly, certain bacteria, notably those of the genus *Xylophilus*, are able to produce and secrete highly pure cellulose through fermentation processes. This form is known as bacterial cellulose (BC) or microbial cellulose [86,87]. In addition, several algal species—such as green algae and red algae—contain algal cellulose [88] within their cell walls, while certain fungi synthesize fungal cellulose [89] as part of their mycelial structures. Although these forms of cellulose share the same chemical structure as plant cellulose, they often exhibit superior properties, including higher purity (free from lignin, hemicellulose, and other impurities), greater crystallinity, higher degree of polymerization, as well as enhanced water retention capacity and mechanical strength.

Considering the microstructure of cellulose, each D-glucopyranose unit (AGU) contains three reactive hydroxyl groups: one primary hydroxyl group at the C-6 position and two secondary hydroxyl groups at the C-2 and C-3 positions. These hydroxyl groups enable cellulose to undergo a range of hydroxyl-related derivatization reactions, including etherification, esterification, acylation, crosslinking, and graft copolymerization [90,91]. Through chemical modification, cellulose’s solubility and stability can be significantly enhanced, thereby imparting it with novel functionalities [91].

Cellulose nanocrystal (CNC) was functionalized by grafting carbon chains to enhance its dispersibility and improve the transfer of its rigidity to less polar matrices, particularly acrylic wood coatings. The modified CNC derivatives exhibited improved dispersion in aqueous acrylic coatings and reduced hydrophilicity. Furthermore, the incorporation of these functionalized nanocellulose fibers helped preserve the aesthetic qualities of the coating while significantly enhancing its abrasion resistance [92]. Similarly, to improve compatibility with the non-polar polymer matrix of acrylic esterified epoxy soybean oil, cellulose nanocrystals (CNCs) were chemically modified and incorporated into a bio-based nanocomposite UV-curing coating system. As the content of modified CNC increases, the hardness, reduced modulus (Er), elastic modulus, and tensile strength of the composite are enhanced, along with a notable improvement in film hardness. Furthermore, the surface modification approach applied to CNC influences the microstructure of the nanocomposite coating, which in turn affects its overall mechanical performance [93]. Virtanen et al. [94] investigated the use of silane-functionalized cellulose nanofibers (CNFs) as an additive in both water-based and solvent-based two-component polyurethane (PU) varnish coatings. The incorporation of silane-functionalized CNF was found to enhance the wear resistance, strength, and elasticity of the PU coatings, while adhesion remained unaffected. The oxygen permeability of the resulting films was significantly influenced by the nature of the dispersion medium (water or organic solvents). Notably, even at low loading levels, silane-functionalized CNFs demonstrated potential as a sustainable alternative to nano-silica for performance enhancement in PU varnish formulations.

### 3.4. Biosynthesis

The enzymatic synthesis of polymers has evolved over several decades, with some of the earliest documented examples, such as the enzymatic synthesis of protein, reported as early as 1930. Particularly, over the past twenty years, this approach has experienced rapid advancement. Compared to conventional chemical synthesis, enzymatic methods offer significant advantages including high specificity, reduced by-product formation, milder reaction conditions, and a reduced environmental footprint. The key objective is the introduction of chemical functionalities onto polymer surfaces while preserving their bulk properties, thereby expanding the scope of advanced applications. Consequently, enzymatic synthesis has been extensively employed in producing a wide range of bio-based materials such as polysaccharides [95], proteins [96], triglycerides [97], and lignin [98]. Commonly utilized enzymes include hydrolases, oxidoreductases, and transferases, with isomerases and cyclases also being used on occasion. The diversity of reaction types encompasses polymer hydrolysis and degradation, polymerization, oxidation, glycosylation, crosslinking, and functional group transformation.

Owing to its mild reaction conditions, utilization of non-toxic and renewable enzyme catalysts, and reliance on sustainable feedstocks, enzymatic polymerization represents a promising pathway for producing bio-based polymers. This approach offers substantial opportunities for advancing green polymer technologies and fostering a more sustainable polymer industry. Ultimately, it is poised to play an essential role in the transition toward—and maintenance of—an environmentally responsible society [99]. The comparison of green chemical approaches highlights the differences between conventional thermochemical methods and enzymatic synthesis of polyesters. Elastomers produced via enzymatic catalysis exhibit superior biodegradability compared to those synthesized through traditional thermocatalytic polyesterification. This enhanced biodegradability underscores the potential of enzymatic strategies to contribute to a closed carbon cycle [100].

However, this approach is also associated with several limitations and challenges:

(1) extended polymerization times are necessary to achieve high molecular weights;
(2) high reaction temperatures (typically between 100 and 140 °C) are often used for the enzymatic synthesis of polymers with high melting points and low solubility, despite a significant reduction in enzymatic activity at such elevated temperatures;
(3) the cost of enzyme catalysts remains relatively high.

The rapid advancement of biotechnology and enzymatic polymerization techniques, coupled with growing recognition of the significant advantages offered by enzymatic processes and bio-based monomers, is expected to drive the commercial production of high-value-added specialty bio-based polymers via biocatalytic routes in the near future. Nevertheless, despite its promise, the large-scale implementation of enzymatic polymerization still faces considerable challenges—particularly in matching the high efficiency and low cost of conventional petroleum-based synthesis pathways [99].

In summary, Table 2 systematically compares the mainstream biomass conversion strategies. This table provides a detailed analysis of the unique advantages and inherent limitations of the four core approaches—direct utilization, physical blending, chemical modification, and biosynthesis—along with their typical application scenarios and representative examples, offering valuable insights for the targeted selection and optimization of conversion processes.

## 4. The Main Bio-Based Coatings

### 4.1. Bio-Based Epoxy Resin Coatings

**Figure 10:**
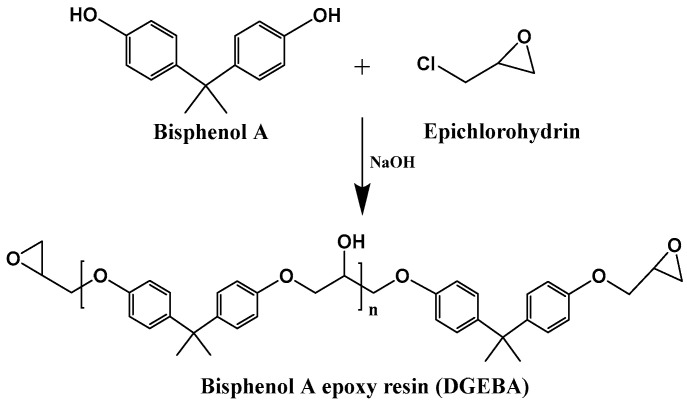
Synthetic route to bisphenol A epoxy resin (DGEBA) [104]. Copyright 2015, Wiley.

Epoxy resins possess an outstanding combination of properties, including excellent mechanical strength, strong adhesion, dimensional stability, as well as high thermal and chemical resistance, making them the dominant material in the coatings market [101]. However, approximately 75% of commercially available epoxy resins are derived from petrochemical resources, with bisphenol A (BPA) serving as the primary precursor for synthesizing bisphenol A diglycidyl ether (DGEBA)-based epoxy resins [102]. Figure 10 shows the synthetic route from bisphenol A to bisphenol A epoxy resin. Studies have indicated that BPA poses serious risks to human health. Due to its phenolic structure, BPA exhibits estrogen-like activity, enabling it to bind to estrogen receptors and disrupt vital bodily functions such as growth, embryonic development, and cell repair, thereby potentially triggering endocrine disorders [101]. Nevertheless, it remains extremely challenging to identify substitutes for DGEBA in contemporary coating applications [103]. In line with the principles of green chemistry and sustainable material development, researchers have been making significant efforts to establish a more environmentally friendly epoxy resin industry through the use of bio-based alternatives.

#### 4.1.1. Plant Oils

As the polymer industry pivots toward more sustainable alternatives, epoxidized plant oils (EVOs) emerged as promising candidates for replacing conventional fossil-derived epoxy systems. Plant oils are one of the renewable resources for fabricating bio-epoxy, which are composed of various triglycerides. The plant oils can be divided into three groups, depending on the degree of unsaturated bonds. Among these oils, linseed oil is used as a drying oil, soybean and canola as semi-drying oils, and castor as a non-drying oil. All of the above have good potential for synthesizing epoxy resins [26]. As shown in Figure 4, usually, the double bonds of triglycerides in plant oils are subjected to ring oxidation to make them suitable for various applications [65].

**Figure 11:**
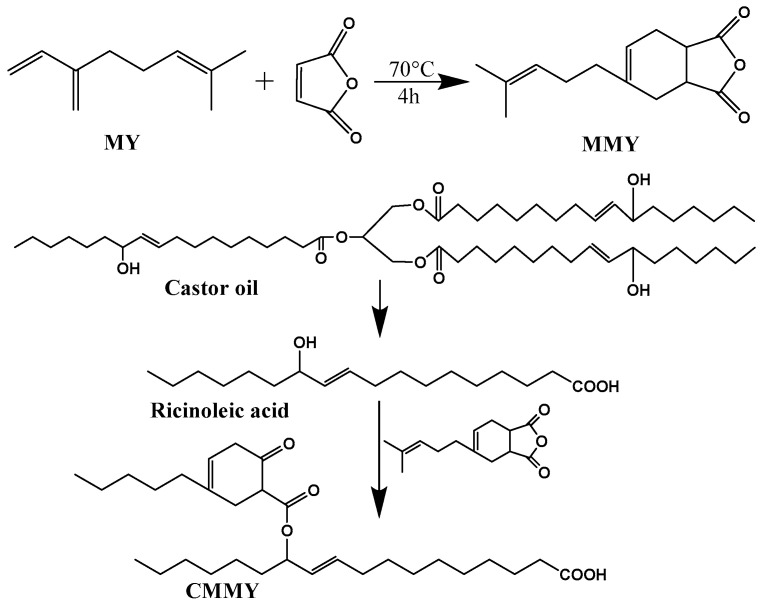
The synthesis route of adduct of myrcene and maleic anhydride (MMY) and adduct of myrcene and maleic anhydride (CMMY) [106]. Copyright 2017, Royal Society of Chemistry.

Plant oils can be converted into epoxy resins via epoxidation [105] and into curing agents via maleinization [105,106]. For instance, epoxy resin has been prepared by epoxidizing linseed oil. Curing agents (H1 and H2) have also been synthesized using linseed oil as the feedstock; specifically, H1 is first obtained through the maleinization of linseed oil, and the subsequent condensation of H1 with diethylenetriamine yields the target curing agent H2. Notably, the composite coating—prepared by incorporating the nanofiller reduced graphene oxide (rGO) into linseed oil-based epoxy resin and the aforementioned curing agent—exhibits excellent anticorrosion performance [105]. Researchers successfully synthesized a series of bio-based epoxy thermosetting resins with varying epoxy-to-hydroxyl group (epoxy:OH) ratios using epoxidized soybean oil and tannic acid, both derived from natural sources. The liquid mixtures were effectively applied and cured on carbon steel substrates using different techniques. Most formulations demonstrated good to excellent anticorrosion protection performance. Samples applied via a spray gun also exhibited satisfactory protective properties, confirming the feasibility of this approach for potential large-scale applications. In summary, these bio-based coatings represent promising green alternatives and are expected to find broad application in protecting various marine carbon steel structures against corrosion [107]. Compared to petroleum-based epoxy resins, those derived from plant oils typically exhibit a lower glass transition temperature (Tg), reduced dielectric constant, and higher water absorption rate [108]. Furthermore, bio-based curing agents (MMY and CMMY) derived from limonene and castor oil were successfully synthesized by Yang (Figure 11). These curing agents were applied to cure epoxy resin E-51. The epoxy resin cured with pure MMY exhibited brittleness, whereas the sample cured with pure CMMY showed excellent flexibility but slightly reduced strength. To address this trade-off, the two curing agents were blended at different weight ratios to develop a novel curing system for E-51 epoxy resin. As the weight ratio of CMMY increased, the tensile strength and glass transition temperature (Tg) of the cured epoxy decreased, while the elongation at break increased significantly. The synthesis of curing agents using bio-based feedstocks helps reduce reliance on petroleum-derived resources. In conclusion, plant oil-based curing agents can be used to cure commercial petroleum-derived epoxy resins. The cured networks demonstrate significantly improved toughness, indicating the potential of these bio-based agents in developing flexible epoxy materials [106].

#### 4.1.2. Plant Phenols

Plant oils are produced in large quantities, are inexpensive, and can be readily transformed into pure epoxy precursors. However, their long aliphatic chains result in excessive flexibility and an undesirably low glass transition temperature (Tg) in crosslinked materials, thereby limiting their technological applicability [109]. To address this limitation, more rigid structures—such as plant-derived phenolic compounds—have attracted growing interest. As a class of secondary metabolites widely present in plants, these compounds feature polyphenolic architectures and are primarily found in bark, roots, leaves, and fruits. Their incorporation into epoxy resins is motivated by the favorable mechanical properties and thermal stability they impart. Consequently, researchers have increasingly focused on the functionalization and synthesis of epoxy resins derived from natural phenolic and polyphenolic structures [110]. Based on the number of phenolic hydroxyl groups, natural polyphenols can be categorized into monophenols (e.g., eugenol, guaiacol, cardanol, vanillin) and polyphenols (e.g., magnolol, gallic acid, protocatechuic acid, lignin).

**Figure 12:**
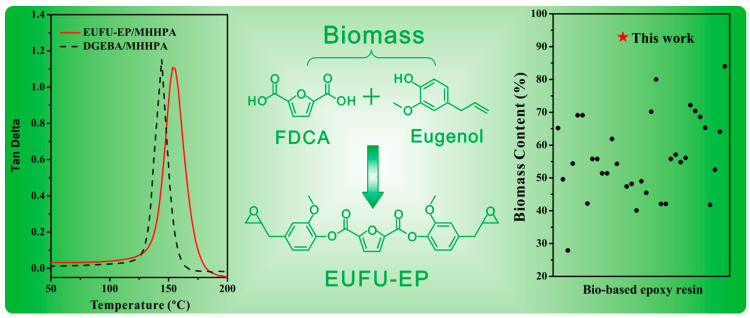
Synthesis rounte of bis(2-methoxy-4-(oxiran-2-ylmethyl)phenyl)furan-2,5-dicarboxylate (EUFU-EP) and tan δ against temperature of EUFU-EP/MHHPA and DGEBA/MHHPA resins and biomass content [111]. Copyright 2017, American Chemical Society.

Eugenol (4-allyl-2-methoxyphenol) is a monophenolic compound that can undergo thiolation to form an epoxy thermosetting resin. This reaction leads to a substantial increase in the glass transition temperature (Tg), which exceeds 100 °C [109]. Furthermore, when reacted with 2,5-furandicarboxylic acid—another biomass-derived building block—a novel epoxy resin with a bio-based content as high as 93.3% was synthesized. This material not only exhibits a markedly higher Tg but also demonstrates competitive mechanical properties and superior flame retardancy (Figure 12). In comparison to conventional petroleum-based epoxy resins, this bio-based alternative shows enhanced overall performance and holds great potential as a sustainable substitute [111].

**Figure 13:**
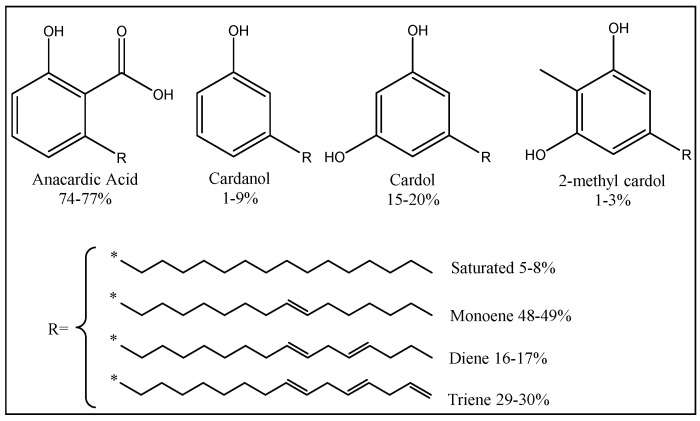
The components of cashew nut shell liquid (CNSL), “*” actually represents the “parent molecular structure to which the R group is attached”—the structural moiety consisting of the benzene ring and the explicitly labeled substituents (e.g., -OH, -COOH, -CH_3_, etc.) on the benzene ring [112]. Copyright 2018, Elsevier.

Among the renewable resource materials, cashew nut shell liquid (CNSL) (Figure 13) is considered as an important starting material due to its unique structural features, abundant availability, and low cost. A large number of chemicals and products have been developed starting from CNSL by taking advantage of the three reactive sites, namely, phenolic hydroxyl, aromatic ring, and unsaturation(s) in the alkenyl side chain. Increasing attention is paid to promising cardanol-based products that could be of potential interest in epoxy resin [112]. Cardanol is produced from CNSL, which could be an interesting substitute to BPA in epoxy resins.

Epoxy resin derived from cashew nut shell liquid (CNSL)—a traditional agricultural byproduct—exhibits superior thermal stability, higher crosslinking density, and an elevated glass transition temperature compared to conventional amine-cured epoxy resins. The resulting single-component coating demonstrates strong adhesion in pull-off tests and excellent corrosion resistance. These properties underscore the potential of CNSL as a sustainable alternative to petrochemical-derived raw materials in epoxy resin production [101]. The commercial epoxidized cardanol derived from cashew nut shell liquid was physically blended with two sucrose-based epoxy derivatives—sorbitol and isosorbide. This combination resulted in a significant increase in both the glass transition temperature and hardness of the blended system [113]. By combining cardanol-based phenolic epoxy resin and phenolic resin with two commercial cardanol-based curing agents, a higher overall bio-based content can be achieved. This increase in bio-content enhances the material’s flexibility and viscoelastic behavior. Such high bio-content epoxy resins represent a more sustainable alternative with reduced environmental impact [114].

**Figure 14:**
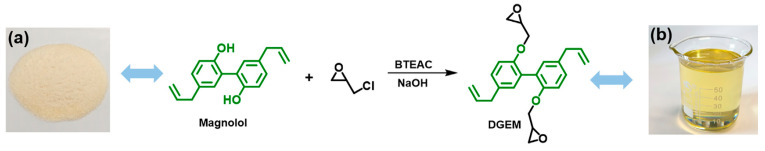
Synthetic route of diglycidyl ether of magnolol (DGEM). (**a**,**b**) are the photographs of magnolol and DGEM [115]. Copyright 2020, Elsevier.

**Figure 15:**
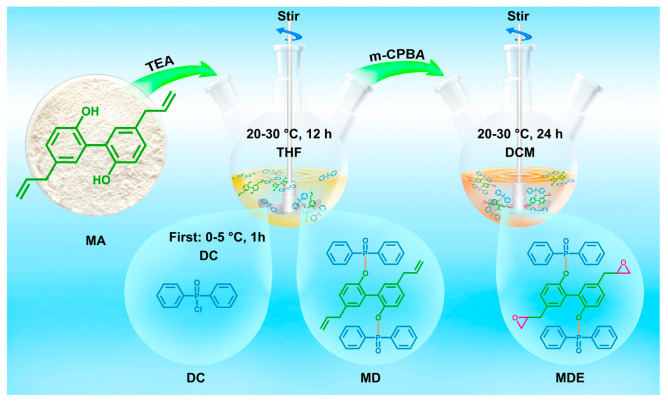
Synthesis process of MD and MDE [116]. Copyright 2023, Elsevier.

Magnolol, a kind of polyphenol, which is extracted from the bark of *Magnolia officinalis*, contains a symmetrical bisphenol and diallyl, which is very suitable for synthesis of various bio-based polymers (Figure 14). Allyl is an active group, which has been widely introduced into various polymers for increasing their crosslink density and improvement of processability [115]. The magnolol-based epoxy resin exhibits intrinsic flame retardancy [115,116], excellent processability [115], outstanding mechanical properties [116], high hydrophobicity [116] and antibacterial property [117]. These attributes offer significant opportunities for developing bio-based epoxy resins that outperform conventional DGEBA-based systems, demonstrating strong potential for use in advanced applications. Figure 14 demonstrates the synthetic route of diglycidyl ether of magnolol (DGEM). Figure 15 expresses the synthesis process of MD and MDE.

Yan and colleagues [118] prepared lignin-based epoxy (LBE) through the etherification of lignin with epichlorohydrin, which was subsequently converted into lignin-based epoxy acrylate (LBEA) via esterification with acrylic acid. The results demonstrated that the incorporation of lignin significantly enhanced the pencil hardness, flexibility, adhesion, chemical resistance, and thermal stability of the epoxy acrylate (EA) coating. Lignin represents a promising reinforcing biomaterial for UV-cured coatings.

**Figure 16:**
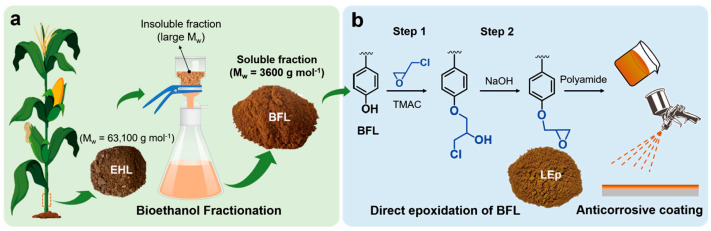
Preparation of lignin-based epoxy resin and anticorrosive coating. (**a**) bioethanol fractionation of enzymatic hydrolysis lignin (EHL) yields bioethanol-fractionated lignin (BFL) and (**b**) direct epoxidation of BFL and preparation of anticorrosive coating [119]. Copyright 2022, Elsevier.

As shown in Figure 16, Wang and co-workers [119] selectively extracted the low molecular weight (MW) fraction of a crop residue-derived enzymatic hydrolysis lignin (EHL) through a bioethanol fractionation process and prepared epoxy resin by direct epoxidation of the bioethanol fractionated lignin (BFL). Subsequently, the BFL was used as a substitute for bisphenol A in preparing lignin-based epoxy resin (LEp) without using additional solvent. The LEp was easily incorporated into the epoxy system by mixing, followed by crosslinking with a polyamide curing agent to fabricate lignin-based epoxy coatings. It was proven that the low MW and high p-hydroxyphenyl content of the BFL offered high solubility and good workability. Lignin-based coatings with 20–40 wt% LEp exhibited good adhesion property (5B) and superior corrosion resistance compared to the commercial epoxy coating. While, with Lep, concentrations increased, (i.e., 60–100 wt%) in coating, the adhesion strength decreased. The coating with 100 wt% LEp still displayed corrosion protection performance comparable to that of the commercial epoxy coating. In conclusion, their study provided a simple and effective approach to converting lignin to epoxy resins for a wide variety of surface coating applications.

In order to address the limitation that the thermal mechanical properties of epoxy soybean oil are unsatisfactory and the flexibility issues when used in thermosetting polymers, Zhen et al. [120] modified lignin by tung oil anhydride (TLs) and then used it as the hardener to compensate for the issue. A fully bio-based epoxy resins possessing a rigid–flexible hybrid structure was fabricated as a result. The tensile strength, dynamic mechanical properties, and thermal properties (Char800) of the thermosets were enhanced due to the lignin used, while the Ea of the curing reaction was increased with the rise in lignin content. They also revealed that the lignin served as a rigid continuous phase and exhibited the ability to impede the curling and sliding of flexible segments, which imparted excellent shape memory properties to the resulting thermosets. In conclusion, a new avenue for the development of high-performance bio-based epoxy resin derived from renewable lignin and ESO was disclosed in this article.

To summarize the above content, vegetable oil-based epoxy resins have a cost advantage due to the abundance of their raw materials, but their glass transition temperature (Tg) is relatively low (typically <80 °C). While plant phenol-modified epoxy resins solve the thermal stability issue (Tg > 100 °C), they face the challenge of complex extraction processes. In the future, it will be necessary to balance performance and cost through “low-cost extraction and molecular structure optimization”.

Compared with bio-based polyurethane coatings, bio-based epoxy resins have better corrosion resistance but poorer flexibility, making them more suitable for harsh environments such as marine corrosion protection rather than flexible substrate coating scenarios.

### 4.2. Bio-Based Polyurethane Coatings

Traditional polyurethane coatings are predominantly derived from petroleum-based raw materials, including polyols (such as polyethers and polyesters) and polyisocyanates (e.g., MDI and TDI). This reliance raises two significant concerns: resource unsustainability and adverse environmental impacts. Consequently, there is growing interest in developing bio-based polyurethane coatings utilizing renewable resources—such as plant oils [121,122], cellulose [123], and lignin [124,125]—as alternative feedstocks. This research direction has gained considerable attention across both academic and industrial sectors, with the aim of reducing the carbon footprint, diminishing dependence on fossil resources, and creating high-performance materials that are either biodegradable or recyclable.

#### 4.2.1. Bio-Based Polyols

The development of bio-based polyurethane centers on replacing petroleum-derived raw materials with alternatives derived from renewable resources. Key substitution pathways include the use of bio-based polyols—as substitutes for conventional petroleum-based polyols—and bio-based isocyanates, which aim to replace traditional petroleum-based variants such as MDI and TDI. Bio-based polyols [126,127,128,129,130] represent the most extensively studied and technologically mature area (in bio-based polyurethane raw materials). As the “soft segments” for bio-based polyurethane synthesis, they dictate fundamental coating properties including flexibility and hydrolysis resistance.

Čuk and co-workers [126] synthesized industrially scalable fully bio-based solvent-free polyester polyols, which were combined with acrylic polyols to prepare high-solid content two-component polyurethane protective coatings for metal surfaces. The results showed that the performance of the bio-based polyols and coatings was comparable to that of their synthetic counterparts. This 100% bio-based and solvent-free polyol can serve as a direct substitute for synthetic polyols, marking a step forward in the green transformation of the coatings industry.

Pan [127] prepared highly functional ester-type and ether-type polyols for polyurethanes (PUs) through the epoxy ring-opening reaction of epoxidized sucrose soyate. The polyurethane coatings made from these polyols exhibit excellent coating hardness and solvent resistance. As new bio-based polyols, sucrose soyate polyols have a high degree of adjustability. Due to their high hydroxyl functionality, they can form polyurethanes with a wide range of crosslinking densities. Thanks to the innovative compact structure of sucrose esters, the viscosities of ester polyols (5–10 × 10^3^ mPa·s) and ether polyols (50–100 × 10^3^ mPa·s) at 100% solid content are acceptable. These polyurethane thermosetting materials have a bio-based content of at least 50%.

**Figure 17:**
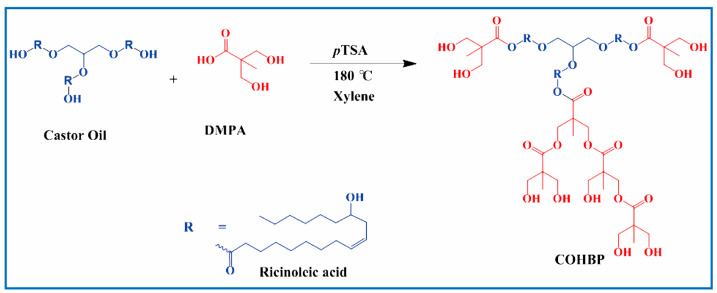
Synthesis of hyperbranched polyol from castor oil [128]. Copyright 2020, Elsevier.

As shown in Figure 17, Patil [128] prepared bio-based hyperbranched polyols (COHBP) using castor oil and dimethylolpropionic acid (DMPA) and used them as raw materials to prepare castor oil-based polyurethanes (PU-COs) and castor oil-based hyperbranched Polyol Polyurethanes (PU-COHBPs). Finally, these polyurethanes were coated on glass plates and low-carbon steel plates. The comprehensive properties of PU-COHBPs were compared with those of PU-COs. It was found that PU-COHBPs exhibited excellent coating properties compared with PU-COs, which was attributed to the high degree of branching, higher degree of polymerization, and better crosslinking density in PU-COHBPs. Therefore, castor oil is one of the best raw materials for synthesizing hyperbranched polyols and their applications in industrial coatings.

#### 4.2.2. Bio-Based Isocyanates

Bio-based isocyanates currently represent a major technical bottleneck in sustainable polyurethane production. As the “hard segments” of polyurethanes, isocyanates (-NCO groups) are critical for imparting key coating properties such as hardness, strength, and abrasion resistance. However, isocyanates are inherently toxic, and prolonged exposure poses significant risks to human health [131,132]. Moreover, their production relies on highly toxic phosgene as a key reactant [132]. To date, large-scale commercialization of bio-based isocyanates remains unrealized, primarily due to high production costs and limited availability of commercial-grade bio-based precursors [133]. Most laboratory-scale studies focus on synthesizing them via phosgenation of bio-based amines derived from sources such as amino acids, vegetable oils, sugars, lignin, and proteins [34]. Nevertheless, the extreme toxicity of phosgene and the complexities involved in purification have severely impeded industrial adoption [131,132].

#### 4.2.3. Non-Isocyanate Polyurethanes

**Figure 18:**
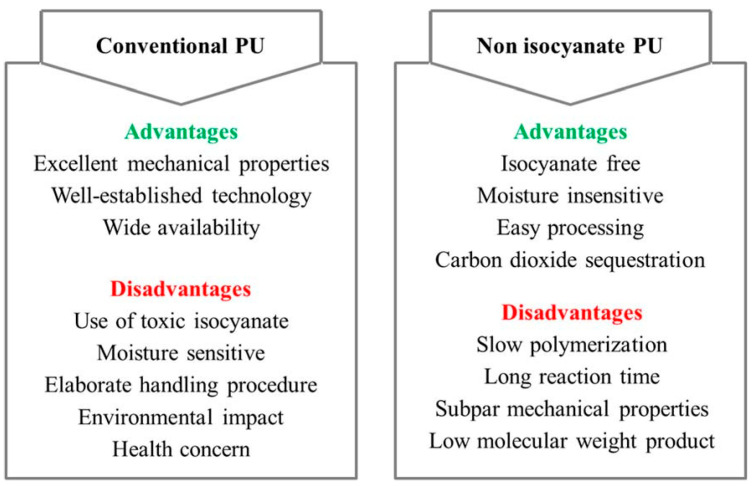
Comparison of conventional PU and non-isocyanate PU [34]. Copyright 2024, Royal Society of Chemistry.

**Figure 19:**
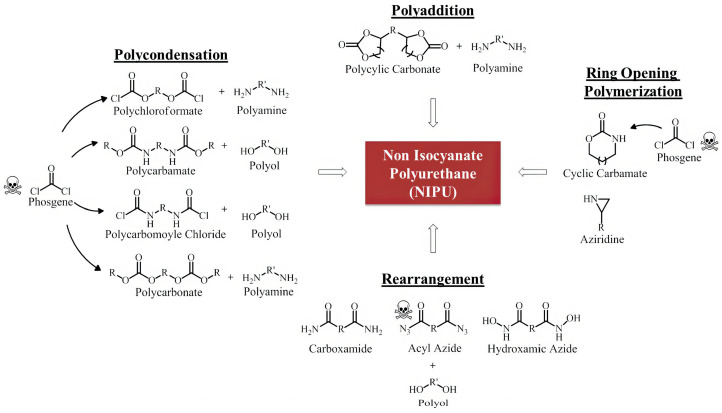
Common synthesis routes of NIPU [34]. Copyright 2024, Royal Society of Chemistry.

A more promising alternative is offered by non-isocyanate polyurethanes (NIPUs), which are typically synthesized through the reaction of cyclic carbonates with polyamines [131,132]. NIPU coatings completely avoid the use of isocyanates and phosgene and exhibit desirable properties such as excellent chemical resistance and low permeability. However, they still underperform compared to conventional polyurethanes in several key areas, including drying time, hardness, elasticity, and water resistance [131]. Figure 18 offers a detailed benchmarking of NIPUs against their traditional counterparts, and Figure 19 depicts the generic synthetic route for NIPUs.

Pichon and colleagues [134] synthesized non-isocyanate poly(urea) urethanes with extremely high bio-based content (>80 wt%) and a controlled amount of reactive hydroxyl end groups via transcarbamoylation reactions. The obtained resins were used as cationic aqueous dispersions in sustainable coating systems. These dispersions were crosslinked with tetramethylol acetylene diurea through their reactive hydroxyl end groups, forming self-supporting films and adhesive layers for textile coatings. These free-standing films exhibit high crosslinking efficiency (gel content > 90%), low water absorption (as low as 2%), high water resistance and methyl ethyl ketone (MEK) resistance (≥200 rubs), along with good mechanical properties (Young’s modulus ranging from 1.5 MPa to 3.1 MPa and elongation at break up to 300%), enabling them to resist fabric deformation when applied to textiles. Finally, when used as adhesive layers on textiles, the coating system ensures good hydrolysis resistance and thermal aging resistance, thus showing promise as a replacement for petroleum-based isocyanate-based adhesive layers.

To address the insufficient environmental friendliness of polyurethane coatings used in aerospace applications, Zareanshahraki and co-workers [135] synthesized a series of sustainable UV-curable non-isocyanate polyurethane acrylate (NIPU-AC) oligomers with different structures and acrylate equivalent weights, which were used as the main building blocks for UV-curable coatings in aerospace. Through rational design of NIPU-ACs, selection of appropriate reactive diluents, and regulation of UV curing conditions, the materials can achieve flexibility at −54 °C (no cracking or delamination at 1/8 inch) and good chemical resistance (MEK double rubs > 90, with no significant change in appearance after contact with relevant fluids). These rapidly curable NIPU coatings have low VOCs and HAPS content and are free of isocyanates, promising to be sustainable alternatives to current polyurethane coatings for aerospace applications.

**Figure 20:**
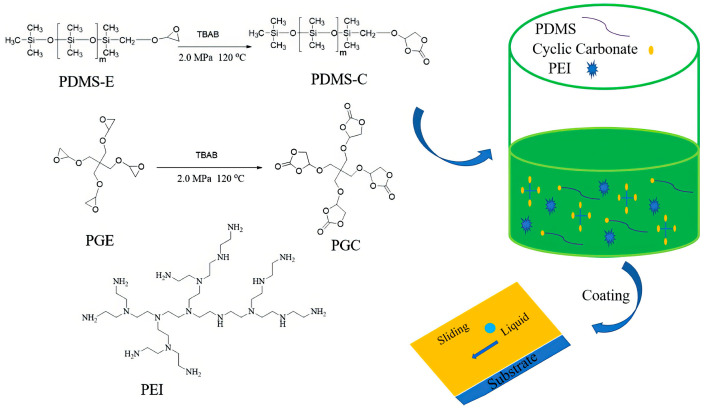
Scheme of synthesis route of the NIPU antismudge coatings and the chemical structures of PDMS-C, PGC, and PEI [136]. Copyright 2022, Elsevier.

As shown in Figure 20, Liu and colleagues [136] developed a novel fluorine-free antifouling non-isocyanate polyurethane (NIPU) coating system by using pentaerythritol glycidyl ether-based cyclic carbonate (PGC)/mono-terminal cyclic carbonate polydimethylsiloxane (PDMS-C) and polyethyleneimine (PEI). PDMS-C was used as a liquid-repellent agent to endow the NIPU coating with low surface energy, while PEI served as a curing agent to form a high crosslinking density in the NIPU coating matrix. The results showed that the NIPU coating with 0.5 wt% PDMS-C added exhibited significant repellency against various liquids (hexadecane, pump oil, water, milk, corn oil, fingerprint fluid, and cola). Dust could slide off with water, and no residual traces of dust or water were left on the coating surface, indicating that the NIPU coating possessed excellent self-cleaning performance. In addition, these NIPU coatings could significantly inhibit ink deposition and still maintain ink resistance after 5000 abrasion cycles, which demonstrated their outstanding mechanical robustness and practical application value. For the NIPU coating containing 0.5 wt% PDMS-C, the water contact angle reaches 118° (compared to 92° for the pure NIPU coating). It maintains over 90% of its antifouling performance after 5000 abrasion cycles, and its MEK rub resistance is ≥200 cycles.

**Figure 21:**
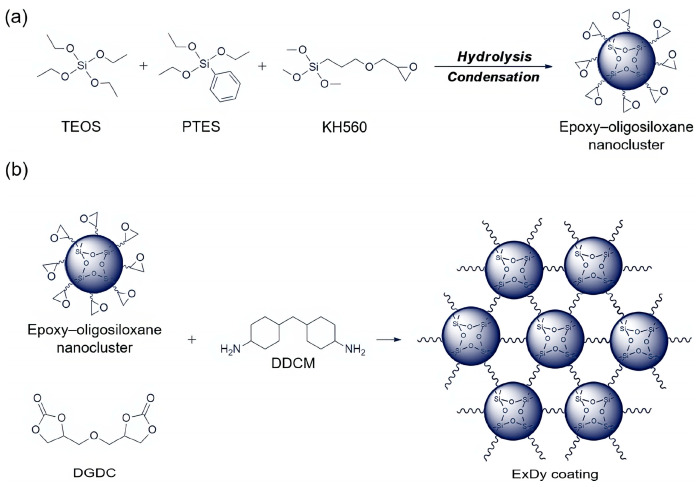
Scheme of (**a**) preparation of the epoxy–oligosiloxane nanocluster and (**b**) schematic representation of the crosslinked network of NIPU coating [137]. Copyright 2023, American Chemical Society.

In Figure 21, Zhang et al. [137] prepared a non-isocyanate polyurethane (NIPU) coating via epoxy-oligosiloxane nanocluster-amine curing reaction and cyclic carbonate-amine polyaddition reaction. This coating exhibits transparency (light transmittance > 92%), hardness (5–7 H), and flexibility (bending diameter of 2 mm). Owing to the high-density polar groups in the NIPU, it demonstrates strong adhesion to various substrates, including aluminum alloy, titanium, steel, glass, ceramics, epoxy resin, and polyethylene terephthalate (PET) (adhesion strength: 2–8 MPa). In addition, the coating can be easily endowed with anti-icing, self-cleaning, and antifouling properties by incorporating amine-terminated polydimethylsiloxane (PDMS) with low surface tension to replace part of the amine curing agent. Notably, since the low-content PDMS tends to enrich on the surface, its introduction has only a slight impact on the mechanical properties of the NIPU coating. This novel coating holds broad application prospects in the fields of foldable devices and marine applications.

Based on the existing research foundation, the future development of bio-based polyurethane coatings will focus on two core directions: First, concentrating on the breakthrough of isocyanate substitution technology—on one hand, addressing the pain points of traditional bio-based isocyanate synthesis, such as its reliance on phosgene and difficulty in large-scale production, to develop brand-new technical routes for preparing bio-based isocyanates via non-phosgene methods; on the other hand, aiming at the performance shortcomings of non-isocyanate polyurethanes (NIPUs), such as long drying time and poor water resistance, expanding the scope of their performance optimization through means like nanocompositing and functional group modification to further enhance practical applicability. Second, promoting the diversification and high-value utilization of raw materials—on the raw material side, further exploring the application potential of low-cost biomass resources such as lignin and agricultural waste to expand the sources of renewable raw materials; on the performance regulation side, realizing the precise customization of coating properties by means of molecular design (e.g., hyperbranched structure construction and copolymerization modification) to meet the differentiated needs of different scenarios such as marine antifouling and flexible electronics.

### 4.3. Bio-Based Alkyd Coatings

Alkyd resins are among the oldest plant oil-derived polymeric resins and are extensively used in the coatings industry. Their widespread application stems from superior performance attributes (including excellent aging resistance, weatherability, heat resistance, and gloss), along with ease of use, low cost, and broad applicability [138].

In traditional alkyd coatings, alkyd resins serve as the primary film-forming component. Conventional formulations rely on three main categories of raw materials: polyols (e.g., glycerol, pentaerythritol, and ethylene glycol), polybasic acids or anhydrides (including phthalic anhydride and adipic acid), and fatty acids or vegetable oils. Among these, only fatty acids/vegetable oils—sourced from plants or animals—qualify as bio-based, while most polyols and polybasic acids remain petroleum-derived.

This reliance on petroleum-based polyols and polybasic acids, however, conflicts with the growing global focus on sustainability, circular economy principles, and carbon footprint reduction. Amid increasingly stringent environmental regulations that limit VOC emissions and fossil resource consumption, traditional alkyd coatings are under growing pressure to transition to greener formulations. Consequently, research efforts have intensified around replacing petroleum-based polyols and polybasic acids with bio-based alternatives, such as glycerol from biodiesel byproducts, succinic acid from fermentation processes, and itaconic acid from renewable feedstocks. These substitutions not only increase the bio-based content of alkyd coatings but also preserve or even enhance their key performance properties, bridging the gap between sustainability and functionality.

According to the Chinese group standard T/CNCIA 01032-2024 [20], this standard specifies that the minimum bio-based content of solvent-based alkyd resin coating products shall be 40%, and that of water-based products shall be 45%.

In contrast, bio-based alkyd coatings represent an advanced, sustainable subclass of alkyd coatings. To qualify as truly “bio-based,” their bio-based content must be increased through targeted raw material substitution, such as:

- replacing petroleum-derived polyols with bio-based alternatives [139];
- substituting petroleum-based polybasic acids with bio-based counterparts [140,141];
- increasing the proportion of plant oils or bio-based fatty acids [142,143].

Teo et al. [139] synthesized novel alkyd resins with short, medium, and long oil lengths via a two-step alcoholysis-polyesterification method using palm kernel oil as the bio-based raw material and glycerol. These alkyd resins are characterized by a molecular weight below 2000 Daltons, a glass transition temperature (Tg) as low as approximately −50 °C, and high thermal stability.

Ghosh and co-workers [140] employed a new and simple strategy to develop an efficient, scalable, low-cost, and green process. Soluble lignin intermediates rich in phenolic hydroxyl and carboxyl groups were extracted from sulfate lignin to replace non-renewable polybasic acids. These intermediates were then used to prepare bio-based alkyd resins, which were further applied to fabricate anticorrosive coatings on metal surfaces. The resulting coatings exhibited a Persoz hardness of 180 s, a gloss of 72 gloss units, and 100% adhesion in the cross-cut test, with no cracks or peeling observed. No corrosion was observed after 120 h of salt spray testing. The characteristics and performance of these coatings are comparable to commercial standards.

Janesch et al. [142] proposed for the first time the use of bio-based dimethyl furan-2,5-dicarboxylate (FDME) to replace petroleum-based phthalic anhydride and prepared bio-based alkyd resins together with vegetable oil triglycerides and glycerol. Several alkyd resins with different FDME contents were synthesized, and various characterization methods showed that with the increase in FDME content, the resin exhibited higher molecular weight and greater viscosity. When the resin with higher molecular weight was coated on glass slides, it could successfully crosslink to form a solid film. The glass transition temperature (Tg) of the prepared coating was approximately 0 °C. This study demonstrated the feasibility of using furanic acid monomers (in this case, dimethyl furan-2,5-dicarboxylate) to convert vegetable oils into resins with potential applications in coatings. Similarly, research on optimizing vegetable oil blending ratios has also provided insights for improving bio-based alkyd resins. Otabor [143] prepared alkyd resins via the polycondensation reaction of monoglycerides with phthalic anhydride by mixing rubber seed oil (RSO) and linseed oil (LSO) in different proportions. These mixed oil-based alkyd resins exhibited good resistance to brine, water, and acids. Compared with the prepared unmixed alkyd samples, an increase in the linseed oil content in the mixture led to a significant decrease in viscosity. The experimental results showed that linseed oil (LSO) possesses excellent properties, making it a high-quality blending oil that can enhance the drying performance of rubber seed oil (RSO). To improve the drying performance of rubber seed oil and thereby synthesize alkyd resins with excellent characteristics, blending rubber seed oil with linseed oil is a feasible method.

Collectively, these studies demonstrate that bio-based alkyd resins can be optimized through diverse strategies—including raw material substitution, process innovation, and oil blending—each addressing specific performance or sustainability targets. It should be noted that only when the modified alkyd coating meets or exceeds these stipulated thresholds—i.e., ≥40% for solvent-borne systems and ≥45% for water-borne systems—can it be officially recognized as “bio-based alkyd coatings”.

Bio-based alkyd coatings have lower production costs than acrylic and epoxy systems, but their lower bio-based content (<45% for waterborne products) and inferior chemical resistance limit their application in high-end coating fields.

### 4.4. Bio-Based Acrylic Coatings

Acrylic coatings have become indispensable in sectors such as architecture, automotive, industrial protection, and woodworking thanks to their excellent weather resistance, outstanding color and gloss retention, superior film-forming capability, and flexible formulation properties. However, their heavy reliance on petroleum-based raw materials in conventional production processes increasingly conflicts with global strategies for sustainable resource use. In recent years, growing attention has been directed toward achieving a green transition by fully or partially replacing petroleum-based feedstocks with renewable biomass resources—such as sugars, glycerol, and lactic acid—for the synthesis of acrylic resins or their monomers. A research team led by Samyn systematically investigated a series of coatings derived from bio-based and fossil-based acrylate monomers and oligomers. Their findings reveal that coatings incorporating bio-based monomers or oligomers exhibit enhanced wear and scratch resistance, along with lower hardness, higher ductility, and improved viscoelastic deformation capacity. Moreover, these bio-based coatings demonstrate higher gloss and hydrophobicity while forming lubricating sliding patches along wear tracks. This study not only confirms that bio-based coatings can effectively enhance mechanical performance but also provides strong evidence for their suitability as protective wood coatings [144]. Thus, vigorously advancing bio-based acrylic coatings represent a promising pathway to synergize high-performance attributes with sustainability goals, thereby contributing to the development of coating systems that integrate environmental friendliness with superior performance.

#### 4.4.1. Synthesis of Bio-Based Acrylic Monomers

**Figure 22:**
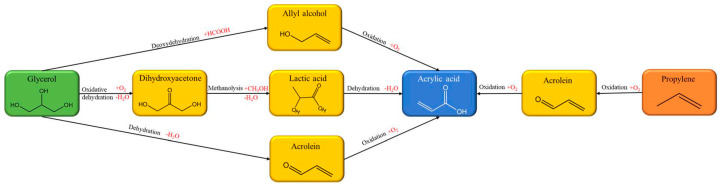
Different pathways to produce acrylic acid from glycerol and propylene [146]. Copyright 2025, Royal Society of Chemistry.

As shown in Figure 22, currently, the synthesis of bio-based acrylic monomers can be achieved through the following basic routes: First, to further expand the high-value utilization pathway of glycerol (a by-product of biodiesel), relying on the structural characteristic of glycerol molecules containing multiple hydroxyl groups, glycerol is first converted into acrolein intermediate via a catalytic dehydration process and then finally converted into acrylic acid through selective oxidation reaction [145]. Second, glycerol reacts with carboxylic acid under specific conditions to generate allyl alcohol intermediate; allyl alcohol is further oxidized to obtain a mixture containing 3-hydroxypropionic acid and acrylic acid; finally, acrylic acid is purified and prepared through the dehydration reaction of 3-hydroxypropionic acid. Third, glycerol undergoes a combination of biocatalysis and chemical conversion: it is first converted into lactic acid via microbial fermentation, and then lactic acid is subjected to catalytic dehydration to eventually obtain acrylic acid [146,147]. The production of bio-based acrylic acid from renewable glycerol not only breaks the reliance on fossil-based raw materials at the source but also achieves a significant reduction in carbon emissions compared to the traditional acrylic acid production via propylene oxidation, thus creating dual advantages in terms of resource sustainability and ecological environmental protection.

Meanwhile, biomass resources such as sugars and starches can be fermented to produce intermediate products like lactic acid or 3-hydroxypropionic acid, which can then be converted into acrylic acid via catalytic dehydration [148].

#### 4.4.2. Construction of High-Performance Bio-Based Acrylic Resins

Owing to its remarkable advantages of high efficiency, energy conservation, and environmental friendliness, photocuring technology is highly aligned with the “high performance” and “sustainability” goals pursued by bio-based materials. This technology can more fully unlock the performance potential of bio-based acrylic resins, thereby accurately meeting the dual requirements of high performance and environmental protection in the modern coating industry. Therefore, in the fields of high-performance bio-based acrylic resin preparation and their coating applications, photocuring technology is undoubtedly a core development direction of great significance and broad prospects.

**Figure 23:**
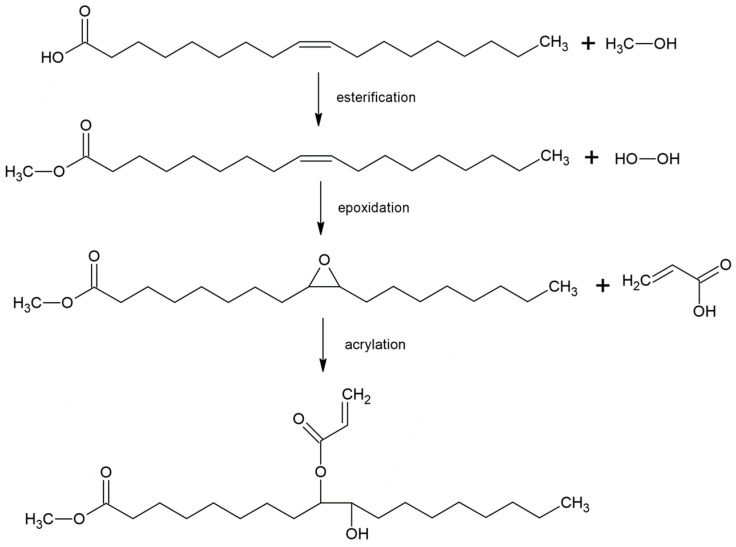
Schematic illustration of acrylated methyl ester of oleic acid (AME-OA) synthesinization [149]. Copyright 2023, Multidisciplinary Digital Publishing Institute.

Kolář and co-workers [149] modified two bio-based raw materials (rapeseed oil and technical-grade oleic acid) to produce two types of bio-based acrylate monomers. Subsequently, they synthesized polymer latex for coating applications via emulsion polymerization—a process involving the copolymerization of methyl methacrylate (MMA), butyl acrylate (BA), and the bio-based acrylate monomers at varying ratios (accounting for 0–20 wt% of the total monomer mixture). Notably, even when the content of bio-based monomers reached as high as 15 wt%, the polymerization still proceeded successfully, achieving high monomer conversion efficiency and low coagulum content, which ultimately yielded latex with long-term stability. Compared with the control acrylic coating (petroleum-based), the latex modified by bio-based monomers exhibited equivalent or even superior film-forming properties in terms of glossiness, adhesion, and water resistance. Additionally, the incorporation of bio-based monomers imparted a plasticizing effect on the coating, making them promising for the industrial production of renewable water-based paints and coating adhesives. These materials serve as suitable alternatives to traditional petroleum-based latex products and can be applied in various coating scenarios, such as decorative or protective coatings for wood, metal, and architectural surfaces. Figure 23 displays the schematic illustration of acrylated methyl ester of oleic acid (AME-OA) synthesinization.

Maity and colleagues [150] used citric acid as the core bio-based feedstock to successfully synthesize a novel UV-curable hyperbranched polyester-urethane-acrylate (PUA) resin, with ester-urethane-acrylate bonds acting as molecular arms. This oligomer was blended with reactive diluents and photoinitiators to formulate a UV-curable system (denoted as UCAP PUA), which was subsequently cured under UV irradiation. Following this, the cured system was applied to the surfaces of wood and metal substrates for coating preparation. The research team conducted systematic characterization on the mechanical, chemical, and thermal properties of the coated substrates, with comparisons made against commercial polyurethane acrylate (denoted as CUA) coatings. The results indicated that in terms of mechanical properties, the UCAP PUA coatings exhibited comparable performance to the commercial CUA coatings in indicators including cross-cut adhesion, pencil hardness, impact strength, and flexibility. Although the UCAP PUA showed slightly inferior chemical resistance and free film properties compared to the commercial product, it demonstrated superior thermal performance (e.g., thermal stability, heat distortion temperature), thereby presenting unique application potential.

Potdar et al. [151] reacted bio-based adipic acid with diethanolamine to form an intermediate, which was subsequently functionalized using glycidyl methacrylate (GMA). Concurrently, a UV-curable bio-based oligomer was synthesized via the ring-opening reaction between epoxidized castor oil and acrylic acid. The as-synthesized reactive diluent and bio-based oligomer were blended at varying ratios to develop a series of bio-based UV-curable formulations, which were then applied onto wood substrate surfaces to fabricate coatings. Characterization results revealed that when the reactive diluent content in the formulation was 40 wt%, the resulting cured coating displayed excellent overall performance: it achieved a 60° gloss of 86, a pencil hardness of 5H, and a cross-cut adhesion of 5B. For thermal properties, its glass transition temperature (Tg) was 81.27 °C, with the maximum thermal decomposition temperature reaching 454 °C. Furthermore, the cured coating exhibited remarkable stain resistance, thereby demonstrating considerable application potential.

Liu et al. [152] synthesized a series of high-functionality acrylates, which featured a triazine ring as the rigid core and long aliphatic chains as the flexible arms. Subsequently, the synthesized acrylates were incorporated into acrylated epoxidized soybean oil (AESO) to prepare UV-curable coatings. Characterization and analysis results indicated that, compared with pure acrylated epoxidized soybean oil (AESO), the introduction of high-functionality acrylates significantly improved the thermal and mechanical properties of the UV-cured films. Further studies revealed that when high-functionality bio-based acrylates were added, the hardness of AESO-based UV-curable coatings was additionally enhanced.

In addition, compared with commercially available petroleum-based acrylates with similar structures, the high-functionality bio-based acrylates not only exhibited a higher bio-based content but also demonstrated more excellent comprehensive performance, thus showing prominent advantages in the field of green coatings.

Wang et al. [153] synthesized acrylated tannic acid (TA-GMA) via the ring-opening reaction between tannic acid (TA) and glycidyl methacrylate (GMA) and employed it as a novel bio-based adhesion promoter. The acrylation modification of tannic acid serves two key functions: on one hand, it significantly enhances the compatibility between tannic acid and UV-curable resins; on the other hand, it enables TA-GMA to participate in the photocuring process, thereby forming a “molecular bridge” between the coating and the metal substrate and strengthening the interfacial bonding. Experimental results demonstrated that TA-GMA could remarkably improve the adhesion strength between the UV-curable coating and the metal substrate. After adding TA-GMA, the pull-off strength of low-carbon steel plates increased from 0.44 MPa to 1.65 MPa, while that of aluminum plates rose from 0.13 MPa to 1.11 MPa; meanwhile, the cross-cut adhesion grade was also significantly improved. Notably, with the substantial optimization of adhesion performance, verification via electrochemical impedance spectroscopy (EIS) tests and salt spray tests revealed that the UV-curable coatings containing TA-GMA also exhibited a simultaneous and significant enhancement in their corrosion protection performance for metal substrates, demonstrating the advantage of synergistic improvement in “adhesion-corrosion resistance”.

In the research and development of high-performance bio-based acrylic resins, the preparation of bio-based acrylate oligomers, bio-based reactive diluents, and bio-based adhesion promoters constitutes a crucial component. These three elements synergistically form the core functional component system of the resin, providing key support for achieving high-performance applications in fields such as coatings.

#### 4.4.3. Optimization and Functional Enhancement of Coating Performance

In the current research and development of bio-based acrylic coatings, it is necessary not only to promote the green transformation of the industry by synthesizing acrylic resins and their monomers through full or partial replacement of petroleum-based raw materials with renewable biomass resources but also to take “surpassing the performance of petroleum-based acrylic coatings” as the core goal. To this end, the directional optimization of coating performance and functional upgrading can be achieved by precisely regulating the molecular structural characteristics of bio-based raw materials, which provides a key pathway for breaking through the performance limitations of coatings.

The research team led by Sung designed and synthesized a novel bio-based acrylate (AECFA) through a series of precise multi-step reactions: first, they utilized the epoxy ring-opening reaction between cardanol glycidyl ether (CGE) and *Crambe abyssinica* oil fatty acid (FACO); next, they carried out epoxidation treatment on the unsaturated chains in the reaction product; finally, they completed the functional modification via acrylation reaction. Subsequently, the team prepared a bio-based acrylate coating by subjecting AECFA to a UV curing process. The chemical structure of AECFA endows it with unique performance advantages—it not only contains flexible aliphatic chains (derived from fatty acids and cardanol) but also possesses rigid aromatic hydrocarbon structures. This characteristic makes its polymer network more tough. Compared with the soybean oil-based acrylate (AESO), the coating not only maintains excellent flexibility but also exhibits superior thermal stability and core coating properties (such as weather resistance and chemical resistance). These results indicate that AECFA, derived from non-food crops, has significant potential in the field of replacing AESO and can be used as a core raw material for the research, development, and production of flexible bio-based acrylate coatings [154].

Demchuk’s research team [155] synthesized latex nanoparticles via the miniemulsion copolymerization process of styrene and methyl methacrylate, using acrylic monomers derived from olive oil and soybean oil as raw materials. In their study, plant oil-based segments were incorporated, aiming to induce a plasticizing effect by reducing the glass transition temperature of the system, thereby regulating the thermomechanical properties of the latex films. The results showed that, compared with the typically rigid characteristics of polystyrene and polymethyl methacrylate, the introduction of bio-based plant oil-based segments significantly enhanced the flexibility of the latex copolymers: this not only improved the film-forming properties of the material but also endowed it with more excellent flexibility and toughness. Additionally, these segments could enhance the hydrophobicity of crosslinked latex films and thus could be used as functional additives to effectively reduce the water sensitivity of the polymer network.

In summary, Table 3 systematically compares the performance characteristics of four main categories of bio-based polymer coatings. This summary table not only clarifies the core advantages and technical bottlenecks of epoxy, polyurethane, alkyd, and acrylic resins but also covers their typical application fields, technological maturity, and cost assessment, thereby providing a clear decision-making basis for material selection in different application scenarios.

### 4.5. Critical Comparison of Major Bio-Based Polymer Coatings

As show in Table 3, different bio-based polymer coatings exhibit distinct characteristics due to variations in raw material sources, synthesis routes, and molecular structures. A comprehensive comparison of their advantages, limitations, and typical application scenarios is crucial for guiding industrial selection and future research directions.

Bio-based epoxy resins: Excel in adhesion, chemical resistance, and mechanical strength, making them ideal for anticorrosion and marine coatings (e.g., CMP NOVA 2000 (Bio) for liquefied ammonia tankers [13,14]). However, plant oil-based epoxy resins suffer from low glass transition temperature (Tg < 80 °C) and poor water resistance, while plant phenol-based counterparts face high extraction costs and complex modification processes [109,119]. The trade-off between performance and cost remains a key bottleneck for large-scale application.

Bio-based polyurethane coatings: Boast flexible formulation and excellent toughness, with non-isocyanate polyurethanes (NIPUs) eliminating toxic isocyanates and phosgene [131]. Nevertheless, bio-based isocyanates are still constrained by high production costs (2–3 times that of petroleum-based isocyanates) and immature non-phosgene synthesis technologies [133]. NIPUs also struggle with long drying times and inferior water resistance compared to traditional polyurethanes [136].

Bio-based alkyd coatings: Offer advantages of low cost, good gloss, and excellent weatherability, with mature industrial production processes [138]. However, their bio-based content is limited by petroleum-derived polyols and polybasic acids, and they require further optimization in terms of drying speed and water resistance [143].

Bio-based acrylic coatings: Demonstrate superior weatherability, color retention, and film-forming properties, suitable for architectural and automotive coatings [144]. The main challenges lie in the high cost of bio-based monomers (e.g., acrylic acid derived from glycerol) and the need to balance rigidity and flexibility in resin design [151].

### 4.6. Intelligent Bio-Based Coatings

The field of bio-based coatings is undergoing a paradigm shift, moving from the substitution of petroleum-based constituents to the design of materials with advanced, intelligent functionalities. This new class of intelligent bio-based coatings comprises smart materials capable of actively sensing and responding to environmental stimuli—including thermal, pH, mechanical, and microbial cues—thereby conferring self-healing, self-cleaning, antimicrobial, and corrosion-reporting properties. These dynamic capabilities significantly augment the longevity, safety, and functional performance of coated substrates.

Exemplifying this trend, renewable feedstocks are being precisely engineered to achieve sophisticated stimulus-responsiveness. Recent work includes the development of dual temperature- and pH-responsive coatings from castor oil-derived monomers [156] and the synthesis of temperature-responsive poly(cholesteryl methacrylate) brushes from cholesterol, which demonstrate reversible transitions and high biocompatibility, underscoring their potential for demanding biomedical applications [157].

Progress in monomer design has also tackled scalability challenges. The strategic manipulation of fatty acid profiles in plant oil-based copolymers enables precise tuning of thermal transitions, facilitating the development of coatings with reliable responsiveness for applications from regulated cell adhesion to low-temperature anti-icing, thereby addressing key performance–cost trade-offs [158].

Notwithstanding these advancements, the integration of complex smart functions into bio-based matrices presents profound challenges, including the preservation of long-term response stability under harsh conditions, the reconciliation of multi-functionality with mechanical robustness, and the establishment of universal performance standards. This review provides a comprehensive summary of these latest developments, critically examining design strategies, operative mechanisms, and emerging applications, and concludes with a forward-looking perspective on the field’s principal challenges and future trajectories

## 5. Other Key Bio-Based Components in Coating Formulations

While bio-based resins constitute the fundamental film-forming matrix, a complete transition to sustainable coatings necessitates the replacement of all ancillary components, including pigments, additives, and solvents, with alternatives derived from renewable resources. The development of these components is crucial for achieving coatings with high bio-based content, reduced toxicity, and a minimized overall environmental footprint.

### 5.1. Bio-Based Pigments and Fillers

#### 5.1.1. Bio-Based Pigments

Bio-based pigments, sourced from plants, microorganisms, and minerals, offer a promising avenue for introducing color in an environmentally benign manner. Unlike conventional inorganic and synthetic organic pigments, their production often involves lower energy consumption and avoids hazardous intermediates.

Significant progress has been made in harnessing microbial fermentation for pigment production. Bacterial genera such as *Streptomyces* and *Serratia* can produce vibrant prodigiosins (red), while fungi are sources of carotenoids (yellow/orange) [159]. Plant-derived colorants, such as anthocyanins (red/blue) and curcumin (yellow), though sometimes limited by lightfastness, are also being explored for specialty coatings [160].

A primary challenge for bio-based pigments is ensuring performance parity with their synthetic counterparts, particularly in terms of color stability, durability, and dispersion stability within polymer matrices. Research is increasingly focused on the nano-encapsulation of natural pigments to enhance their stability and on using metabolic engineering to optimize microbial strains for higher pigment yield and color intensity [161].

#### 5.1.2. Bio-Based Fillers

Fillers are an important component of coatings. They not only improve the mechanical properties, anticorrosion properties, and weather resistance of coatings but also, in the form of special fillers, endow coatings with special functions such as antibacterial activity and electrical conductivity. With the increasing urgency of green development needs, the development of low-cost bio-based fillers is of great significance for promoting the sustainable development of the coating industry. Among them, lignin and cellulose are abundant in nature, and a large amount of waste containing these two components is generated during industrial and agricultural production; their wide sources and low cost make them ideal raw materials for preparing bio-based fillers.

As described in Section 3.3 “Chemical Modification”, Song and their teammates [85] prepared colloidal lignin micro-nanospheres (LMNSs) and used them as multifunctional bio-based fillers for waterborne wood coating (WBC). The incorporation of LMNSs into WBC enhanced the strength and toughness of the polymer matrix, highlighted the color and texture of wood, and improved the discoloration resistance of wood coated with WBC. Ma et al. [162] prepared lignin micro-nanospheres (LMNSs) and applied them to fabricate functional superhydrophobic coatings on wood surfaces, with a contact angle (CA) of 164.4° and a sliding angle (SA) of 5°. After a series of tests including tape peeling, water droplet impact, corrosive liquid immersion, and organic solvent immersion, the superhydrophobic coating still maintained excellent superhydrophobic properties, particularly exhibiting strong organic solvent resistance. In addition, the coating also showed a significant photothermal effect, which facilitates its application in efficient deicing and antibacterial scenarios.

As mentioned earlier, to improve the dispersibility of CNC and CNF, their surfaces can be functionalized via chemical modification. When these modified materials are used as bio-based fillers in coating preparation, they not only significantly enhance their dispersion stability in the resin matrix but also simultaneously improve the wear resistance of the coating film [92,94] and strengthen key mechanical properties of the film, such as strength and elasticity [93,94], thus achieving the synergistic optimization of the coating’s comprehensive performance.

Examples of applications for other bio-based fillers include the following: Wang et al. [163] used bamboo as the raw material and, through a combination of thermochemical treatment and mechanical grinding processes, prepared renewable bio-based ultrafine bamboo charcoal particles (UFBCs). They then used these particles as a substitute component for zinc particles in zinc-rich epoxy coatings, successfully developing a zinc-based epoxy coating with a low zinc content (22 wt%). Test results show that the corrosion resistance of this coating is significantly improved, and this enhancement stems from the synergistic effect between zinc particles and UFBC particles, which is specifically reflected in two aspects: On one hand, the particle size of UFBC particles is smaller than that of zinc particles, enabling them to effectively fill the gaps between zinc powder and the cured epoxy matrix, thereby reducing the number and size of internal defects in the coating and minimizing the penetration channels for corrosive media. On the other hand, the surface of UFBC particles is rich in functional groups such as hydroxyl and carboxyl groups. These functional groups not only improve the dispersion stability of zinc powder in waterborne epoxy resins but also optimize the uniform distribution of zinc powder in the coating film, thereby enhancing the efficiency of zinc powder as a sacrificial anode-type corrosion inhibitor. In addition, the partial replacement of zinc powder with UFBC not only significantly reduces the raw material cost of zinc-based epoxy coatings but also reduces the potential environmental pollution caused by high-zinc-content coatings, achieving a balance between economic efficiency and environmental friendliness.

### 5.2. Bio-Based Additives

Additives are indispensable for tailoring the processing and application properties of coatings. The development of bio-based alternatives is a vibrant area of research aimed at replacing petroleum-derived plasticizers, dispersants, and stabilizers.

Traditional phthalate plasticizers are being superseded by safer, renewable alternatives. Epoxidized vegetable oils (e.g., epoxidized soybean oil) and citrate esters (e.g., acetyl tributyl citrate) have proven effective in providing flexibility to various polymer resins while demonstrating lower toxicity and improved biodegradability [164].

Lignin, a complex polyphenol, has emerged as a highly versatile bio-based additive. Its inherent structure allows it to function as a potent UV-absorber and antioxidant, significantly enhancing the weatherability of coatings [78,79,80]. Furthermore, biosurfactants like sophorolipids and rhamnolipids, produced by yeasts and bacteria, are being investigated as effective bio-based dispersants and wetting agents for stabilizing pigment particles in waterborne systems [165].

### 5.3. Bio-Based Solvents

The substitution of volatile organic compound (VOC)-emitting solvents is critical for reducing the environmental impact of coatings. Bio-based solvents, typically characterized by low toxicity and high biodegradability, are gaining prominence.

Lactate esters, such as ethyl lactate, are excellent green solvents derived from fermentative lactic acid. They offer good solvating power for a wide range of resins and are readily biodegradable [166]. Similarly, bio-based alcohols (e.g., bio-ethanol) and esters derived from them are being integrated into coating formulations. Terpenes, such as d-limonene from citrus peels, are also utilized as effective bio-based solvents for resins and cleaning applications [167].

The adoption of these solvents is not only driven by their performance but also by favorable life-cycle assessments (LCAs) that demonstrate a substantial reduction in carbon emissions compared to petroleum-based solvents like toluene or xylene [168].

In conclusion, the advancement of bio-based pigments and fillers, additives, and solvents is integral to the holistic sustainability of the coatings industry. While significant innovations are underway, these components are less commercially mature than bio-based resins. Future research should focus on enhancing their performance, ensuring cost-competitiveness, and improving compatibility with bio-based resins.

## 6. Current Challenges and Future Outlook

As an important development direction in the field of green chemical industry, bio-based coatings have shown significant potential in promoting the process of sustainable development. However, their current development path still faces many challenges and limitations, which restrict the further breakthrough of their industrialization and marketization.

### 6.1. The Main Current Challenges and Limitations

#### 6.1.1. Technical and Performance Bottlenecks

Dilemma in Balancing Performance and Cost: At present, some bio-based coatings still struggle to fully match the highly mature petroleum-based products in terms of key performance indicators such as long-term durability, substrate adhesion, and resistance to chemical media corrosion. Meanwhile, their complex bioconversion processes and limited industrial scale result in significantly higher production costs than traditional coatings, which restricts their market competitiveness.

Inherent Limitations of Raw Material Supply: The acquisition of biomass raw materials is constrained by geographical and climatic conditions, posing risks to supply stability. Furthermore, the complex chemical composition of natural products leads to insufficient uniformity in the composition and performance of raw materials across different batches, presenting severe challenges to the consistent quality control of end products.

#### 6.1.2. Industrialization and Market Barriers

Incomplete Industrial Chain Structure: A complete industrial chain—from large-scale collection and efficient pretreatment of biomass raw materials to the synthesis of high-performance coating products—has not yet been effectively connected. Taking bulk biomass such as lignin and cellulose as examples, the technical routes for their high-value conversion remain immature, resulting in extremely low practical utilization rates.

Insufficient Market Awareness and Acceptance: Currently, the market share of bio-based coatings in the global coatings market is less than 5%, and the sector as a whole is in the initial stage of market introduction. End-users have limited awareness of the long-term performance reliability and comprehensive benefits of bio-based coatings, and a market trust system still needs to be established.

#### 6.1.3. Lagging Development of Standard Systems and Certification Frameworks

Lack of Uniformity in Basic Definitions and Testing Standards: There is currently no globally unified consensus on the scientific definition of “bio-based” materials, leading to the coexistence of multiple systems for testing key indicators such as bio-based carbon content. Systematic differences between testing methods represented by the American ASTM D 6866-24a standard [17] and the European EN 16640 standard [19] pose dual challenges for enterprises in terms of technical adaptation and compliance costs when addressing market access requirements in different regions.

Insufficient Adaptability of Performance Evaluation Standards: Existing coating performance evaluation standards are primarily based on traditional petroleum-based systems, making it difficult to comprehensively and scientifically reflect the unique properties of bio-based coatings. In areas such as biodegradability, differentiated aging mechanisms, and gas barrier properties, there is a lack of targeted evaluation methods and standard support. This not only affects the accurate market positioning and promotion of products but also substantially restricts the diversity of technological research and development and the progress of innovation.

Technically, the “imbalance between performance and cost” is the core bottleneck (e.g., the cost of bio-based isocyanates is 2–3 times that of petroleum-based ones). Non-technically, the lack of standards leads to chaos in market access. Both issues need to be addressed simultaneously to accelerate industrialization.

### 6.2. Future Outlook of Bio-Based Coatings

Despite existing challenges, bio-based coatings are poised for rapid development driven by technological innovation and policy support. Future research should focus on the following specific directions:

#### 6.2.1. Precise Design of High-Performance Bio-Based Resins

Develop multi-component copolymerization technologies to balance the performance of bio-based resins (e.g., combining plant oil-based flexibility with plant phenol-based rigidity) and achieve targeted optimization of key properties such as Tg, water resistance, and mechanical strength.

Explore novel bio-based monomers (e.g., furan derivatives, lignin-derived phenols) and develop efficient synthesis routes to improve bio-based content while reducing production costs. For example, further optimize the synthesis process of 2,5-furandicarboxylic acid (FDCA)-based epoxy resins to enhance scalability

#### 6.2.2. Breakthroughs in Key Auxiliary Component Technologies

Accelerate the development of bio-based isocyanates with non-phosgene synthesis routes, focusing on reducing catalyst costs and improving reaction efficiency to achieve cost parity with petroleum-based isocyanates.

Develop high-performance bio-based pigments and additives (e.g., UV-absorbers derived from lignin, biosurfactants) with good compatibility and stability, realizing the full bio-based transformation of coating formulations.

#### 6.2.3. Advancement of Advanced Application Technologies

Promote the development of intelligent bio-based coatings, such as self-healing coatings based on dynamic covalent bonds and stimulus-responsive antifouling coatings, to expand application scenarios in high-end fields such as aerospace and flexible electronics.

Optimize coating application technologies (e.g., UV curing, electrostatic spraying) to improve film formation quality and reduce energy consumption, adapting to the characteristics of bio-based resins.

#### 6.2.4. Improvement of Industrial Chain and Standard Systems

Establish a stable biomass raw material supply chain and develop multi-source complementary raw material systems (e.g., combining crop-based and agricultural waste-based biomass) to address supply instability.

Promote the unification of global bio-based content testing standards (e.g., aligning ASTM D 6866-24a [17] and EN 16640 [19]) and establish targeted performance evaluation systems for bio-based coatings (e.g., biodegradability and aging resistance testing methods).

#### 6.2.5. Integration of Circular Economy Concepts

Develop degradable and recyclable bio-based coating systems to realize the closed-loop utilization of coating waste, such as designing bio-based coatings that can be degraded into biomass raw materials under specific conditions.

Explore the high-value utilization of industrial by-products (e.g., lignin from papermaking, glycerol from biodiesel) to reduce the environmental impact of raw material production.

## 7. Conclusions

This review has systematically charted the landscape of bio-based coatings, underscoring their critical role in the transition toward sustainable materials. The analysis confirms that strategies for biomass conversion—from direct utilization and physical blending to chemical modification and biosynthesis—provide a versatile toolkit for creating functional coatings, signaling a field that has matured beyond simple fossil fuel substitution to pioneer advanced material systems. This evolution is demonstrated by the development of high-performance epoxy, polyurethane, alkyd, and acrylic resins, complemented by innovations in bio-based pigments, additives, and green solvents. However, persistent challenges—namely the performance–cost imbalance, vulnerabilities in biomass supply chains, and the lack of unified global standards—continue to hinder widespread commercialization. Overcoming these barriers requires a paradigm shift from isolated technological advances to an integrated, system-level approach. The future mainstream adoption of bio-based coatings therefore hinges on a holistic strategy that synergizes cutting-edge research, industrial scaling, and supportive policymaking. Through such collaborative efforts, bio-based coatings can fully realize their potential as high-performance, sustainable material systems capable of meeting global industry demands while contributing meaningfully to environmental preservation and a circular economy.

This review differentiates itself from prior work by offering a holistic and critical perspective on bio-based coatings. It moves beyond the typical focus on isolated resin systems by providing a systematic comparison of four major types—epoxy, polyurethane, alkyd, and acrylic—and seamlessly incorporates them with bio-based pigments, additives, and solvents within a unified “resin-auxiliary-application” framework. A significant innovation lies in the inclusion of the latest advancements in intelligent and biomedical coatings, which points to new, high-value application avenues. Conclusively, this review articulates a cohesive “closed-loop industrial chain” strategy that synergizes raw material sustainability, technological innovation, and standardized policies, presenting a comprehensive reference for accelerating industrial adoption.

## Figures and Tables

**Table 1:** Comparison of characteristics of bio-based materials derived from different raw materials.

Raw Material Category	Representative Source	Core Advantage	Inherent Defect	Typical Coating Types
Plant source	Plant oils (linseed oil, soybean oil, canola oil, castor oil et al.) Plant phenols (eugenol, guaiacol, cardanol, vanillin, magnolol, gallic acid, protocatechuic acid, lignin) Polysaccharides (starch, cellulose, pectin, inulin, glycogen, sorbitol, isosorbitol, hyaluronic acid, chondroitin sulfate, xanthan gum, dextran, lentinan, ganoderma lucidum polysaccharide, agar, sodium alginate) Rosin (turpentine, terpenes, terpineol, resin acids) Biological acids (itaconic acid, citric acid, amino acid, rosin acid, lactic acid, oxalic acid, phytic acid, tartaric acid, fatty acids) Bio-based non-isocyanate polyurethane (epoxidized plant oils, sugar derivatives, lignocellulose derivatives)	Abundant sources; widely present in nature; true renewability; low toxicity/VOC potential for any sources.	Plant oils: oxidation instability during storage, affecting long-term performance. Plant phenols: complex extraction processes lead to high costs. Polysaccharides: poor water resistance of films formed. Biological acids: some have strong acidity, potentially corroding substrates. Bio-based non-isocyanate polyurethane: harsh synthesis conditions and low reaction efficiency. Performance variability: chemical composition can vary with crop, season, and climate, leading to batch-to-batch inconsistencies. Compatibility issues: some bio-based polymers may have poor compatibility with traditional petroleum-based resins or additives.	Alkyd coatings, epoxy coatings, polyurethane coatings, acrylic coatings, benzoxazine coatings plant acid-modified functional coatings
Animal source	Chitin, chitosan, collagen, gelatin, shellac, beeswax	Strong antibacterial property; good biocompatibility; excellent film-forming ability; excellent adhesion and film toughness for some (e.g., collagen, gelatin).	Limited by animal breeding, supply stability is challenged. Beeswax: low melting point, prone to softening at high temperatures. Long microbial fermentation cycle and strict control requirements for fermentation conditions (temperature, pH, dissolved oxygen, etc.), making large-scale production costly. Some microbial-derived polymers: insufficient mechanical strength for high-strength coating applications. Ethical concerns and market acceptance issues related to animal-derived ingredients. Potential allergenicity for certain animal-derived proteins (e.g., shellac, collagen).	Shellac-based protective coatings, chitosan-modified anticorrosive coatings, beeswax-based wood coatings
Microbial source	Chlorella, spirulina, *Dunaliella*, *Haematococcus pluvialis*, xanthan gum, gellan gum, bacterial cellulose, polyhydroxyalkanoates	Precise regulation of structure; excellent weather resistance; biodegradable; environmental friendliness. Non-competition with arable land: cultivation in fermenters does not require farmland. Utilization of waste streams: many microbes can use industrial/agricultural waste as culture media.	High production cost associated with fermentation and downstream processing. Relatively low yield for some target polymers.	Xanthan gum-based thickened coatings, polyhydroxyalkanoates-based biodegradable coatings, bacterial cellulose-based high-performance coatings polylactic acid (PLA)-based coatings

**Table 2:** Comparison of biomass conversion strategies.

Conversion Strategy	Advantages	Limitations	Application Scope	Representative Examples	References
Direct utilization	Simple process, low cost; retains natural properties of biomass	Poor performance (e.g., long drying time of tung oil); limited application	Wood protection, fruit preservation coatings	Tung oil wood coating, beeswax fruit coating	[46,53]
Physical blending	Low research and development cost; flexible property adjustment; compatible with traditional resins	Poor miscibility; limited performance improvement	Anticorrosion, flame-retardant coatings	Cardanol/DGEBA blend anticorrosion coating	[58,60]
Chemical modification	Precise molecular regulation; significant performance enhancement	Complex process; high cost; potential environmental impact of chemical reagents	High-performance coatings (e.g., high Tg, corrosion resistance)	Epoxidized plant oil epoxy resin, lignin-modified acrylic resin	[67,85]
Biosynthesis	Mild reaction conditions; high specificity; low environmental footprint	Long reaction time; high enzyme cost; difficulty in large-scale production	Biodegradable coatings, specialty coatings	Enzymatically synthesized polyhydroxy-alkanoate (PHA) coatings	[99,100]

**Table 3:** Comparative analysis of key bio-based polymer coating systems.

Coating Type	Key Advantages	Core Limitation and Liabilities	Typical Applications	Technological Maturity	Relative Cost and Feasibility	References
Bio-based epoxy	Excellent adhesion and chemical resistance High mechanical strength and hardness Good thermal stability (especially with phenolics) good corrosion protection	Low Tg and flexibility (plant oil-based) Complex extraction (plant phenol-based) Often requires petro-based curing agents Brittleness if not properly formulated	Marine and automotive anticorrosion coatings High-performance industrial floors Electronic encapsulants	Commercial (growing)	Medium to high Plant oils: cost-competitive Plant phenols: more expensive	[105,111,119]
Bio-based polyurethane (PU)	Superior abrasion resistance and toughness Excellent flexibility and low-temperature performance NIPUs avoid toxic isocyanates	High cost and toxicity of isocyanates (conventional route) Poor hydrolytic stability (some polyols) NIPUs: slower curing and lower performance than conventional PUs	Furniture and wood finishes Automotive interior coatings Textile and leather coatings Aerospace coatings	Commercial (mature for polyols)	Medium to high Bio-polyols: competitive Bio-isocyanates/NIPUs: premium	[128,136,137]
Bio-based alkyd	Low cost and easy application Good penetration and wetting on substrates Autoxidative curing (air-drying) Proven technology, easy to retrofit	High VOC (in solvent-borne forms) Slow drying compared to acrylics/PUs Susceptible to yellowing and oxidative degradation Limited chemical/alkali resistance	Architectural and decorative paints Primer and undercoats for metal/wood Heavy-duty maintenance coatings	Commercial (mature)	Low to medium Most cost-effective bio-based option	[139,142,143]
Bio-based acrylic	Outstanding UV and weather resistance High clarity and color retention Fast curing (especially UV-curable) Good mechanical property balance	High rigidity and brittleness (if not modified) Lower chemical resistance than epoxies/PUs Reliance on fossil-based acrylic acid (partially)	Clear varnishes for wood and plastic Overprint varnishes and inks Architectural and automotive topcoats	Research and development to commercial (growing fast)	Medium Bio-acrylic acid: currently premium	[150,151,154]

## Data Availability

No new data were created or analyzed in this study.
